# PLK1 inhibition exhibits strong anti-tumoral activity in *CCND1*-driven breast cancer metastases with acquired palbociclib resistance

**DOI:** 10.1038/s41467-020-17697-1

**Published:** 2020-08-13

**Authors:** Elodie Montaudon, Joanna Nikitorowicz-Buniak, Laura Sourd, Ludivine Morisset, Rania El Botty, Léa Huguet, Ahmed Dahmani, Pierre Painsec, Fariba Nemati, Sophie Vacher, Walid Chemlali, Julien Masliah-Planchon, Sophie Château-Joubert, Camilla Rega, Mariana Ferreira Leal, Nikiana Simigdala, Sunil Pancholi, Ricardo Ribas, André Nicolas, Didier Meseure, Anne Vincent-Salomon, Cécile Reyes, Audrey Rapinat, David Gentien, Thibaut Larcher, Mylène Bohec, Sylvain Baulande, Virginie Bernard, Didier Decaudin, Florence Coussy, Muriel Le Romancer, Guillaume Dutertre, Zakia Tariq, Paul Cottu, Keltouma Driouch, Ivan Bièche, Lesley-Ann Martin, Elisabetta Marangoni

**Affiliations:** 1 Translational Research Department, Institut Curie, 26 Rue d’Ulm, 75005 Paris, France; 2 Institute of Cancer Research, 123 Old Brompton Road, SW7 3RP London, UK; 3 Department of Genetics, Institut Curie, Paris, France; 4 Alfort Veterinary School, 7 Av. Du Général-de-Gaulle, 94000 Maisons-Alfort, France; 5 Department of Pathology, Institut Curie, Paris, France; 6 INRA, APEX-PAnTher, Oniris, Rue De La Géraudière Cedex 3, 44322 Nantes, France; 7 Genomics of Excellence (ICGex) Platform, Institut Curie Research Center, Paris, France; 8 Department of Medical Oncology, Institut Curie, Paris, France; 9 Inserm U1052, Centre de Recherche en Cancérologie de Lyon, 28 Rue Laennec, 69000 Lyon, France; 10 Department of Surgery, Institut Curie, Paris, France

**Keywords:** Cancer models, Targeted therapies, Breast cancer

## Abstract

A significant proportion of patients with oestrogen receptor (ER) positive breast cancers (BC) develop resistance to endocrine treatments (ET) and relapse with metastatic disease. Here we perform whole exome sequencing and gene expression analysis of matched primary breast tumours and bone metastasis-derived patient-derived xenografts (PDX). Transcriptomic analyses reveal enrichment of the G2/M checkpoint and up-regulation of *Polo-like kinase 1 (PLK1)* in PDX. PLK1 inhibition results in tumour shrinkage in highly proliferating *CCND1*-driven PDX, including different RB-positive PDX with acquired palbociclib resistance. Mechanistic studies in endocrine resistant cell lines, suggest an ER-independent function of PLK1 in regulating cell proliferation. Finally, in two independent clinical cohorts of ER positive BC, we find a strong association between high expression of *PLK1* and a shorter metastases-free survival and poor response to anastrozole. In conclusion, our findings support clinical development of PLK1 inhibitors in patients with advanced *CCND1*-driven BC, including patients progressing on palbociclib treatment.

## Abstract (web summary)

Identifying novel therapies for the treatment of CDK4/6 inhibitor-resistant patients is of great importance. Here, the authors demonstrate that PLK1 inhibition is a potential therapeutic target in *CCND1*-driven and in RB-positive Palbociclib-resistant breast cancers.

## Introduction

Oestrogen receptor-positive BC accounts for over 80% of primary breast malignancies^1^. Classically, ER-positive BC patients are treated with ET which block ER signalling. Although the introduction of ET has significantly increased survival, resistance remains a significant problem^2^.

A major challenge for the successful treatment of ER-positive BC has been the identification of new therapeutic targets for endocrine-resistant disease. Inhibitors of mTOR and cyclin-dependent kinases 4 and 6 (CDK4/6) substantially improve progression-free survival^3^ and are now standard of care for the treatment of advanced ER + BC. However, the mTOR inhibitor everolimus is poorly tolerated and intrinsic or acquired resistance to CDK4/6 inhibitors are frequent events, limiting the success of these treatments^4,5^. Resistance to CDK4/6 inhibitors is emerging as a critical consideration in patients care and clinical drug development. Identifying novel therapies for the treatment of CDK4/6 inhibitor‐resistant patients is of great importance.

Bone is the most common metastatic site in ER+ patients^6^, however, bone metastases are technically challenging to biopsy and analyse. Difficulties concern both tumour tissue acquisition and techniques for analysis and DNA/RNA extractions. Patient-derived xenografts of bone metastases have not been reported yet.

Here, we establish PDX models from bone metastatic biopsies of patients progressing on ET and treated by vertebroplasty. PDX models are analysed at the genomic and transcriptomic level and compared to patient’s early primary tumours to identify new therapeutic targets associated with endocrine resistance in the metastatic setting. Transcriptomics analysis show enrichment of G2/M cell cycle signalling with an increased expression of several targetable kinases. In particular, *PLK1*, a serine threonine kinase, which plays an essential role in centrosome maturation, mitotic chromosome segregation and mitosis, is among the top up-regulated genes in PDX models. Based on this finding we hypothesised that PLK1 could represent a therapeutic target in endocrine resistant advanced BC. We show that PLK1 inhibition results in dramatic tumour shrinkage in highly proliferating *CCND1*-driven PDX including different RB-positive PDX with acquired palbociclib resistance.

## Results

### PDX of bone metastases show activation of the G2/M checkpoint

To identify new therapeutic targets in endocrine resistant metastatic BC, we established PDX models from metastatic bone biopsies taken from ER-positive BC patients treated by vertebroplasty. Seven PDXs were initially established. The clinical characteristics of the corresponding patients are summarised in Table 1. Six out of seven ER-positive BC patients were progressing on ET (aromatase inhibitors (AI), tamoxifen or fulvestrant). HBCx-176 and HBCx-180 were recently established from patients progressing on palbociclib plus letrozole.

**Table 1:** Characteristics of patients and PDX.

PDX ID	Patient’s primary tumour	Treatment before vertebroplasty	MFS (years)	Bone met. and PDX IHC	PDX			
					Mutations	Gene amplification	Gains	Homozygous deletion
HBCx-118	ER+ PR− HER2-Ki67 NA	FEC, Vinorelbine, Tamoxifen, Letrozole, Paclitaxel, Capecitabine	7	ER+ PR+	*BRCA2* *AKT1*			
HBCx-124	ER+ PR+ HER2-KI67 50%	AC	0.5	ER+ PR+		*FGFR1* *CCND1* *MYC, CCNE2*	*AURKA*	
HBCx-131	ER+ PR+ HER2-Ki67 NA	Cyclophosphamide + Epirubicine + Docetaxel, AC, FUN, Tamoxifen, Anastrozole, Letrozole	10	ER+ PR+		*FGFR1* *CCND1*		*CDKN2A/B*
HBCx-134	ER+ PR+ HER2-Ki67 60%	PI3Kα inhibitor, Letrozole, FEC + Docetaxel	1	ER+ PR−	*PIK3CA*	*CCND1, ESR1*	*HRAS*	*CDKN2A/B*
HBCx-137	ER+ PR+ HER2-Ki67 35%	FEC as adjuvant followed by letrozole	0	ER+ PR−	*GATA3*	*CCND1, FGFR1, FGFR2, PAK1, TERT*	*CCNE2*	
HBCx-139	ER+ PR+ HER2-Ki67 NA	FEC, Tamoxifen + Triptorelin,Docetaxel, Capecitabine, Cyclophosphamide, Paclitaxel, Letrozole, Exemestane + Everolimus, Doxorubicin, Fulvestrant	9	ER+ PR+	*PIK3CA*	*PAK1*	*CCND1* *CCNB1* *CDK7* *CCNE2*	
HBCx-142	ER + PR-HER2-Ki67 NA	FEC, Tamoxifen, Triptorelin, Paclitaxel, Anastrozole, Capecitabine, Eribulin	13	ER+ PR−	*AKT1* *MTOR*			
HBCx-180	ER+ PR−	FEC, Tamoxifen, palbociclib + letrozole	14	ER+ PR−			*CCND1*	
HBCx-176	ER+ PR−	Palbociclib + letrozole	14	ER−	*NF1*			

Characteristics of patients and PDX: FEC: 5-FU epirubicine cyclophosphamide. *AC* adriamycin cyclophosphamide, *FUN* 5FU-Navelbine, *MFS* metastasis-free survival.

PDXs were molecularly characterised at the genomic level by a targeted next generation sequencing (NGS) analysis of 95 genes (the most frequently mutated genes in BC^7^). Copy number alterations (CNA) were predicted from whole-exome sequencing or by Cytoscan HD array. Significantly mutated genes and CNA in putative cancer drivers genes are shown in Table 1. Details on mutations and variant frequencies are shown in Supplementary Data 1 and CNA in PDX tumour samples are listed in Supplementary Data 2.

**Fig. 1:**
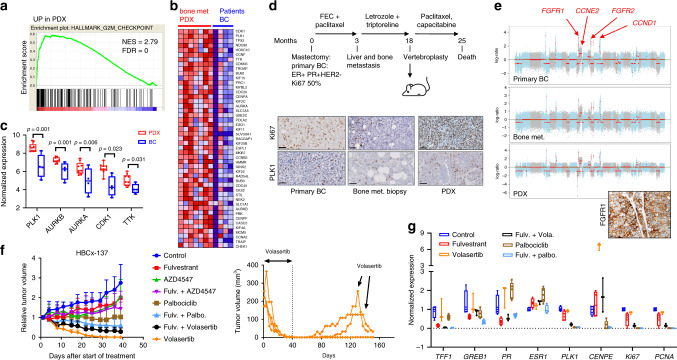
Up-regulation of mitotic genes in PDX established from bone metastases and response to PLK1 inhibition in the HBCx-137 PDX. **a** GSEA enrichment plots corresponding to hallmarks G2/M checkpoint. The analysis was performed on RNA from seven PDX and four primary breast tumours. NES normalised enrichment score, FDR: false discovery rate. **b** Heat map of gene expression changes in the G2 M hallmark. **c** Up-regulation of mitotic genes transcripts in bone metastasis derived PDX (*n* = 9) as compared to patient’s primary tumours (*n* = 4) (min to max whiskers plots with line indicating the median). **d** Clinical history of the patient corresponding to HBCx-137 PDX. The patient was diagnosed with ER + PR + breast cancer highly proliferating (Ki67 35%) at 52 year old. Lung and bone metastases were detected only 3 months after the start of adjuvant chemotherapy. She was treated by letrozole and triptorelin before vertebroplasty and PDX establishment. Immunohistochemistry analysis of Ki67 and PLK1 in primary breast tumour, bone metastasis and PDX (Ki67 *H*-score were 35%, 10% and 95% respectively, and PLK1 *H*-score were 6, 15 and 25%). Scale bar 50 μm. **e** Genomic profile of primary tumour, bone metastasis and PDX assessed by whole-exome sequencing. The three tumours carry focal amplification of *FGFR1*, *FGFR2* and *CCND1* and CN gain of *CCNE2*. FGFR1 expression has been validated by IHC in the HBCx-137 PDX (95% of positive cells). **f** In vivo targeting of HBCx-137 PDX by volasertib (10 mg/kg 4 times/week), palbociclib (75 mg/kg 5 days/week) and AZD4547 (12.5 mg/kg 5 days per week) in monotherapy and in combination with fulvestrant. *n* = 5 independent xenografts (control, fulvestrant, AZD4547, fulv. + AZD4547), *n* = 7 (volasertib), *n* = 8 (fulv.+vola), *n* = 9 (palbo, palbo + fulv.). Mean ± SD. Volasertib treated xenografts were monitored after end of treatment to detect tumour recurrence. Two xenografts relapsed at day 70 and 100 and were rechallenged with volasertib. **g** RT-PCR analysis of ER regulated genes (*TFF1*, *GREB1* and *PR*), *ESR1*, *PLK1*, *Ki67*, *PCNA* and *CENPE* in treated tumours. Min-to-max whisker box plots with line indicating the median, *n* = 4 independent xenografts for all groups, with the exception of volasertib where *n* = 3 (xenografts were sacrificed before complete response for these xenografts). Source data from **c** and **g** are provided as a Source Data file.

To identify activated signalling pathways in bone metastasis, we performed comparative transcriptomic analysis of the bone metastasis derived PDX compared to the patients’ primary BC. Gene set enrichment analysis (GSEA) showed augmentation of different hallmarks associated with cell proliferation and G2/M DNA damage checkpoint (E2F target, G2/M checkpoint, DNA damage, and mitotic spindle) in PDX as compared to primary tumours (Supplementary Table 1). The most significantly enriched gene sets in primary tumours were epithelial mesenchymal transition (EMT), NFKB/TNF, TGF beta signalling, and gene sets associated with the loss of human stroma (inflammatory response and interferon response). The integrative pathway and network analysis are shown in Supplementary Fig. 1. Figure 1a, b shows the hallmark of the G2/M checkpoint and mitotic spindle with the heat map of the top differentially expressed genes in the G2/M checkpoint gene set. Notably, different targetable kinases associated to mitosis were up-regulated in PDX, including *PLK1*, *AURKA*, *AURKB*, *CDK1* and *MPS1* (Fig. 1c), and *PLK1* was among the top up-regulated genes in PDX models.

### PLK1 is a therapeutic target in highly proliferating PDX models

Based on these findings, we hypothesised that *PLK1* could represent a therapeutic target in PDX of metastatic BC. Therefore, we assessed the efficacy of the PLK1 inhibitor volasertib (BI 6727)^8^ in the HBCx-137 PDX, established from an aggressive ER + BC (patient’s clinical history is detailed in Fig. 1d and associated figure’s legend). Immunohistochemical analysis revealed a strong expression of PLK1 and Ki67 in the PDX, the patient’s bone metastasis and primary breast tumour. The CNA profile of the primary breast tumour, the bone metastasis and the PDX, were highly similar with focal amplification of *FGFR1*, *FGFR2* and *CCND1* and CN gains of *CCNE2* (Fig. 1e, Supplementary Data 2). Focal amplification of *FGFR1* also associated with high protein expression in PDX. The in vivo anti-tumour activity of volasertib was assessed in comparison with the CDK4/6 inhibitor palbociclib and the FGFR1/2/3 inhibitor AZD4547^9^, as monotherapies or in combination with fulvestrant. Strikingly, volasertib caused rapid tumour shrinkage and complete response achieved in 4 weeks (Fig. 1f). The combination of volasertib with fulvestrant was less efficient than volasertib alone, although tumour regression was observed in all xenografts. Statistical analysis of tumour growth inhibitions are depicted in Supplementary Data 3. Within the same time frame, treatment by palbociclib alone or combined with fulvestrant resulted in stable disease. Two volasertib-treated xenografts relapsed 6 weeks after end of treatment but responded to a second round of treatment with volasertib resulting in rapid tumour shrinkage, indicative of tumour rewiring (Fig. 1f).

We hypothesised that PLK1 inhibition may influence ER ligand-dependent and/or ligand-independent transcription, as it was previously suggested in vitro using BC cell lines^10,11^. We therefore analysed the expression of the ER-regulated genes (ERGs) *TFF1*, *GREB1* and *PR*, together with *PLK1*, *Ki67*, *PCNA* and *CENPE* (a centromere associated protein that accumulates in G2 phase and is involved in chromosome alignement^12,13^), in treated tumours. No significant alteration in the expression changes of the ERG was evident in volasertib-treated xenografts. Contrastingly, the expression of *CENPE* was significantly increased (Fig. 1g). Interestingly, the expression of *PLK1* was decreased by fulvestrant. The expression of *Ki67* and *PCNA* was unchanged in volasertib treated xenografts, in contrast to palbociclib-treated xenografts (Supplementary Data 4). These results suggest that the anti-tumour activity of volasertib in this tumour is ER independent and not associated with a decreased proliferation but rather with a cell cycle arrest in the G2 phase, consistent with its known mode of action^8^.

**Fig. 2:**
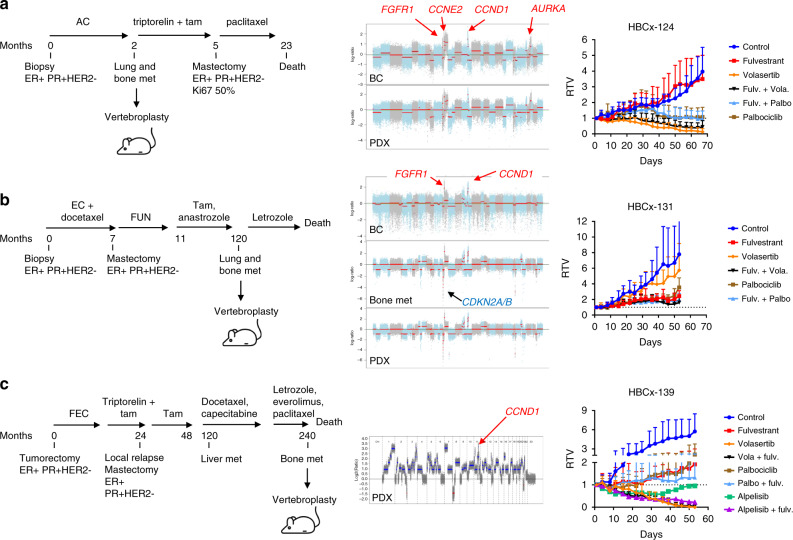
Response to volasertib in *CCND1*-driven PDX. **a** clinical history, genomic profile and drug response of PDX HBCx-124. The HBCx-124 PDX was established from a patient diagnosed with metastatic BC at 26 years old. She was treated by chemotherapy (AC) and tamoxifen was given after the vertebroplasty. Copy number alterations in patients’ BC and PDX include amplification of *CCND1*, *CCNE2*, *AURKA* and *FGFR1*. The PDX was treated by volasertib as compared to palbociclib ± fulvestrant. RTV = relative tumour volume. Mean ± SD (*n* = 5 in control and fulvestrant treated groups; *n* = 7 in fulv. + vola., palbo, and palbo + fulv. treated groups; *n* = 8 in volasertib treated group). **b** Clinical history, genomic profile and drug response of the HBCx-131 PDX. The PDX was established from a patient diagnosed with breast cancer at 41 years old. The patient was treated by neo-adjuvant chemotherapy followed by mastectomy and adjuvant hormone-therapy (tamoxifen + anastrozole). She recurred with lung and bone metastasis 10 years later and was treated by letrozole. Patient’s tumour, bone met. and PDX carry amplification of *CCND1* and *FGFR1*. A homozygous deletion of *CDKN2A/B* emerged in bone metastasis (and PDX).The PDX was treated by volasertib as compared to palbociclib ± fulvestrant. Mean ± SD (*n* = 6). **c** Clinical history, CNA profile and drug response of PDX HBCx-139. The PDX was established from a patient diagnosed with breast cancer at 33 years old, treated by neo-adjuvant chemotherapy and adjuvant tamoxifen. She recurred 10 years later with liver and bone metastases and was treated by different lines of chemotherapies and everolimus. The genomic profile of HBCx-139 PDX was analysed by cytoscan HD array: the PDX carry amplification of *PAK1* and has CN gains for *CCND1*, *CDK7* and *CCNB1*. The PDX is also *PIK3CA* mutated and was treated by volasertib as compared to palbociclib and the PI3Kα inhibitor alpelisib (BYL-719) ± fulvestrant. RTV relative tumour volume. Mean ± SD (*n* = 5 in control group, *n* = 6 in fulvestrant, palbo treated group, *n* = 7 in volasertib, volasertib + fulv., alpelisib and alpelisib + fulv.).

To further define the subset of tumours susceptible to respond to PLK1 inhibition, we tested the anti-tumour activity of volasertib as compared to palbociclib in three additional PDX with amplification or CN gain of *CCND1*. PDX HBCx-124 was established from a young patient diagnosed with metastatic BC at 26 years old. Patient’s breast tumour and bone metastasis-derived PDX show amplification of *FGFR1*, *MYC*, *CCNE2*, *CCND1* and *AURKA* (Fig. 2a). The PDX was resistant to fulvestrant, responded to volasertib with complete response and to palbociclib (±fulvestrant) with stable disease. PDX HBCx-131 was established from a patient diagnosed with BC at 41 years old who relapsed 10 years after mastectomy (Fig. 2b). Patient’s breast tumour, bone metastasis and PDX showed amplification of *FGFR1* and *CCND1* and bone metastasis and PDX carry a homozygous deletion of *CDKN2A*. In this tumour, inhibition of PLK1 and CDK4/6 did not arrest tumour growth. Finally, PDX HBCx-139 was established from a patient diagnosed with BC at 33 years old, who recurred 9 years after mastectomy and adjuvant hormone-therapy (Fig. 2c). The PDX showed amplification of *PAK1*, CN gains in *CCND1*, *CCNB1*, *CDK7* and *CCNE2* and a hotspot mutation of *PIK3CA*. The PDX responded to volasertib and to alpelisib (PI3Kα inhibitor) + fulvestrant with complete response and to palbociclib + fulvestrant with stable disease.

Finally, we analysed the response to volasertib as compared to AKT1 and mTOR inhibitors in two PDX with *AKT1* and *mTOR* mutations and no amplification of *CCND1:* HBCx-118 and HBCx-142 (Supplementary Fig. 2; Supplementary Data 3). The clinical history of patients was similar: both have been treated by neo-adjuvant chemotherapy followed by tamoxifen (adjuvant setting) and letrozole (HBCx-118) or anastrozole (HBCx-142) (metastatic setting), prior to vertebroplasty and PDX establishment. Both PDXs were resistant to fulvestrant. PDX HBCx-118 (*BRCA2* and *AKT1* mutated) responded to the combination of AZD5363 plus fulvestrant, while PDX HBCx-142 (*AKT1* and *mTOR* mutated) responded with tumour regression to everolimus and with stable disease to volasertib, AZD5363 and palbo + fulvestrant.

In summary, targeting PLK1 lead to a striking tumour regression in three out of fpur *CCND1*-driven PDX models, while *AKT1* and *mTOR* mutated PDX preferably responded to *AKT1* and *mTOR* inhibitors.

### PLK1 is a therapeutic target in PDX with acquired palbociclib resistance

**Fig. 3:**
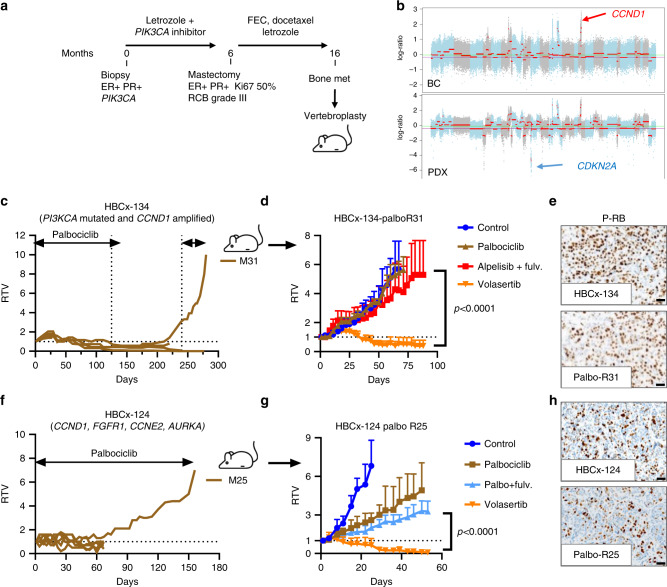
Reponse to volasertib in *CCND1*-driven PDX models with acquired resistance to palbociclib. **a** Clinical history of the patient corresponding to HBCx-134 PDX: the patient’s tumour carries a *PIK3CA* mutation and the patient was treated by a PI3Kα inhibitor + an aromatase inhibitor in the neo-adjuvant setting. At mastectomy the pathological analysis classified the tumour as RCB-III (resistance to neo-adjuvant treatment). The patient was treated by adjuvant chemotherapy (FEC, 5-FU, epirubicin, cyclophosphamide and docetaxel) and letrozole before metastatic recurrence and PDX establishment. **b** Genomic profile (WES) of patient’s tumour and PDX model showing amplification of *CCND1* and *CDKN2A* homozygous deletion in the PDX. **c** Establishment of a PDX with acquired resistance to palbociclib from the HBCx-134 model: 4 HBCx-134 xenografts were treated by palbociclib during 4 months: all xenografts responded. Treatment was stopped and mice were followed-up: xenograft #°31 relapsed 4 months after end of treatment and exhibited resistance when retreated by palbociclib. **d** The PDX was re-engrafted in another set of mice and treated by palbociclib to confirm resistance, by volasertib and by the PIK3CA inhibitor alpelisib (BYL-719) + fulvestrant, during 3 months. Results confirmed palbociclib resistance and revealed a cross-resistance to alpelisib + fulvestrant. RTV relative tumour volume. Mean ± SD. *n* = 4 xenografts/group. *P* ≤ 0.0001 (Mann–Whitney *t* test, two-sided). **e** Immunohistochemical analysis of phospho-RB in HBCx-134 and HBCx-134 palbo-R PDX (70% of phospho-RB positive cells in both tumours). Scale bar is 50 μm. **f** Establishment of a PDX with acquired resistance to palbociclib from a HBCx-124 xenograft that was treated by palbociclib during 5 months: tumour growth of xenograft n°25 was stabilised during the first 2 months and increased on treatment until day 150. **g** Tumour response to palbociclib alone or combined with fulvestrant and volasertib in the HBCx-124 palbo-R25 PDX. RTV = relative tumour volume. Mean ± SD. *n* = 5 in control, palbo, palbo+ fulv.; *n* = 8 in volasertib group. *P* ≤ 0.0001 (Mann–Whitney *t* test, two-sided). **h** Immunohistochemical analysis of phospho-RB in HBCx-124 and HBCx-124 palbo-R PDX (38% and 36% of phospho-RB positive cells, respectively).

Despite the clinical benefit of CDK4/6 inhibitors, tumour resistance develops in most patients in the metastatic setting and finding new therapeutic targets for these patients remains an unmet clinical need. We therefore developed two PDX models of acquired resistance to palbociclib: the first PDX was derived from the HBCx-134 PDX, established from the bone metastasis of a 71-year-old patient. The patient’s breast tumour carried a *PIK3CA* mutation (also found in the PDX) and the patient was treated with a PI3Kα inhibitor combined with the AI letrozole in the neo-adjuvant setting (Fig. 3a). After pathological examination the residual tumour was classified as RCB-III (no response to neo-adjuvant treatment). Patients’ and PDX tumours both carried *CCND1* amplification and a homozygous deletion of *CDKN2A* was identified in PDX (Fig. 3b). HBCx-134 xenografts which initially responded to palbociclib (Fig. 3c), were exposed long term to CDK4/6 inhibition in order to establish a palbo-resistant PDX. Xenograft Palbo R31 showed cross-resistance to alpelisib plus fulvestrant but was highly responsive to volasertib (tumour growth inhibition of 96%; *p* < 0.0001, Mann–Whitney test) (Fig. 3d). Genomic profiling and a NGS analysis of 571 cancer associated genes revealed no differences in CN (Supplementary Fig. 3) and mutation profile between the parental and the palbo-resistant PDX: with both carrying a mutation in *PIK3CA* (His1047Arg) and a *TP53* mutation (Arg248Trp). Abundance of phospho-RB was also similar in the parental and palbociclib resistant xenografts indicating that palbociclib resistance was not due to RB loss in this model (Fig. 3e). The second model of palbociclib acquired resistance (Palbo-R25) was established from a HBCx-124 xenograft that was exposed to palbociclib treatment for 5 months (Fig. 3f): tumour growth was stabilised during the first 2 months prior to tumour escape. HBCx-124 Palbo-R25, re-implanted in another set of mice, showed a striking response to volasertib (Fig. 3g) with tumour regression observed in all xenografts. By contrast to the parental HBCx-124 PDX that responded to palbociclib and palbociclib + fulvestrant with stable disease (Fig. 2a), HBCx-124 Palbo-R25 xenografts progressed on palbociclib + fulvestrant treatment. As for the HBCx-134 PDX, we did not find differences in copy number (Supplementary Fig. 3) nor in the mutational profile of the 571 genes analysed and phospho-RB expression was conserved in palbo-R25 PDX (Fig. 3h).

**Fig. 4:**
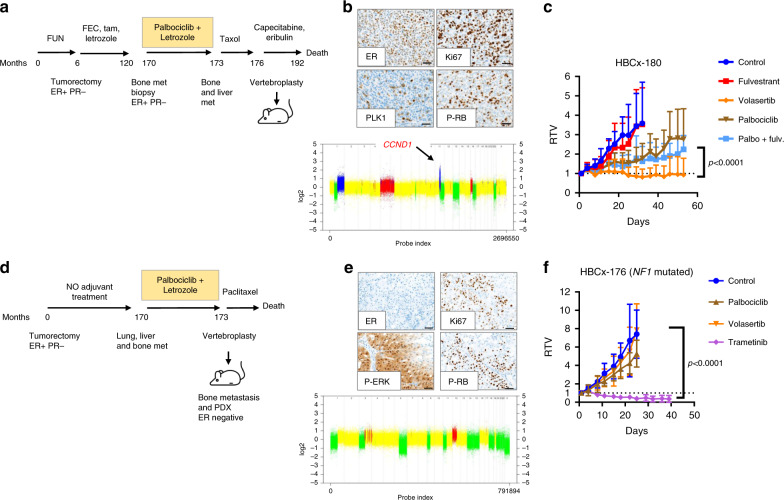
Response to treatment of PDX established from breast cancer patients progressing on palbociclib treatment. **a** Clinical history of the patient corresponding to HBCx-180 PDX: the patient was treated by neo-adjuvant chemotherapy and adjuvant chemotherapy + endocrine treatment (tamoxifen and letrozole). The patient recurred with bone metastases 14 years after diagnosis and was treated by palbociclib + letrozole during 3 months followed by paclitaxel before vertebroplasty and PDX establishment. **b** Immunohistochemical analysis of Ki67 (54% of positive cells), PLK1 (10% of positive cells), ER (95% of positive cells) and phospho-RB (37% of positive cells) in PDX HBCx-180 and copy number profile (Cytoscan HD array). **c** Response to fulvestrant, palbociclib, palbociclib and fulvestrant and volasertib in PDX HBCx-180. Mean ± SD (*n* = 5 fulvestrant treated group; *n* = 6 in the other groups). *P* ≤ 0.0001 (Mann–Whitney *t* test, volasertib versus palbo + fulv., two-sided). **d** Clinical history of the patient corresponding to HBCx-176 PDX: the breast tumour was removed by tumorectomy and was ER+ PR−. The patient did not received adjuvant endocrine treatment, recurred with bone, liver and lung metastases 14 years after diagnosis and was treated by palbociclib + letrozole during 3 months before vertebroplasty and PDX establishment. **e** IHC analysis of ER (0% of positive cells), Ki67 (54% of positive cells), P-ERK (70% of positive cells) and P-RB (47% of positive cells) and copy number profile (Cytoscan HD array). **f** Response to trametinib, palbociclib and volasertib in PDX HBCx-180. Mean ± SD; *n* = 7 for control, palbociclib, volasertib, *n* = 8 in trametinib group. *P* ≤ 0.0001 (Mann–Whitney *t* test, volasertib versus palbo + fulv.).

Finally, we tested the efficacy of volasertib in two PDX models established from patients progressing on palbociclib + letrozole treatment. Patient corresponding to PDX HBCx-180 recurred with bone metastases 14 years after breast surgery and 4 years after end of adjuvant endocrine therapy. She received three cycles of palbociclib + letrozole followed by paclitaxel, before vertebroplasty and PDX establishment (Fig. 4a). The genomic and IHC analyses of the PDX revealed an amplification of *CCND1* (Supplementary Data 2), a mutation of *PIK3CA* (Supplementary Data 1), high expression of Ki67 and PLK1 and positivity of phospho-RB (Fig. 4b). The PDX reproduced resistance to palbociclib ± fulvestrant and responded to volasertib with stable disease (Fig. 4c).

PDX HBCx-176 was established from a patient who relapsed with metastatic disease 14 years breast surgery and was treated with palbociclib+ letrozole during 3 months (Fig. 4d). Both bone metastasis and PDX tumours were ER negative. The PDX carried a frameshift insertion in the *NF1* gene (Supplementary Data 1), was positive for P-RB and exhibited strong expression of P-ERK, a marker of ERK-MAPK signalling activation (Fig. 4e). HBCx-176 responded to the MEK inhibitor trametinib with tumour regression and was resistant to both palbociclib and volasertib (Fig. 4f).

Overall, these results demonstrate that targeting PLK1 is an effective treatment in *CCND1*-driven PDX models with acquired resistance to palbociclib, including PDX established from patients pre-exposed to palbociclib. In addition, we show that a *NF1*-mutated tumour is resistant to both palbociclib and volasertib but respond to the MEK inhibitor trametinib.

### Response to volasertib is correlated with high expression of PLK1 and Ki67

**Fig. 5:**
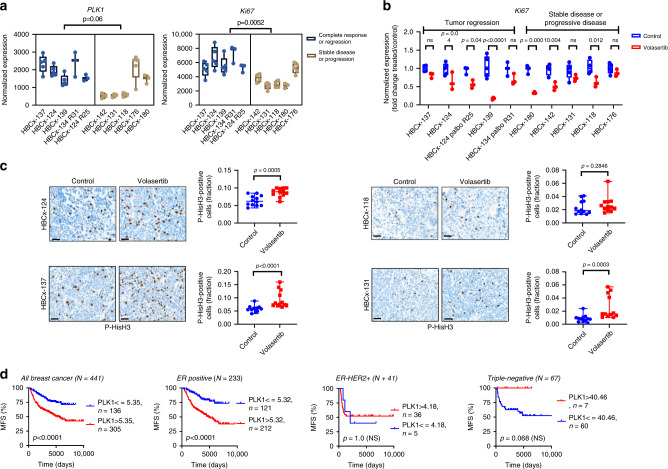
Analysis of proliferation markers in PDX and prognostic value of PLK1 expression in early ER + BC. **a** RT-PCR analysis of *PLK1* and *Ki67* gene expression in control xenografts. Data are presented as a min/max Whiskers plots with line indicating the median. *n* = 5 for HBCx-137, HBCx-139, HBCx124 and HBCx-131. *n* = 4 for HBCx-118 and HBCx-142. *n* = 3 for HBCx-134 palbo-R. (Nested *t* test, two-sided) **b***Ki67* gene expression in control and treated PDX samples (fold changes treated/control). Data are presented as a Min/Max Whiskers plots with lines indicating the median. *n* = 3 for all volasertib-treated xenografts and control xenografts from HBCx-124 palboR25, HBCx-134 palboR31 and HBCx-180; *n* = 4 control HBCx-137 and HBCx-118 xenografts, *n* = 5 for control xenografts of the other PDX. (Sidak’s multiple comparisons test) **c** Analysis of phospho-histone H3 (Ser10) expression by immunohistochemistry in control and volasertib-treated xenografts (harvested 24 h after a single treatment). Scale bar 50 μM. Representative picture of *n* = 3 xenografts/group. For each tumour four tissue sections were analysed. Scatter plots (median with range). (Mann–Whitney *t* test, two-sided) **d** Metastasis-free survival (MFS) curves of patient groups from the series of 441 breast tumours according to *PLK1* mRNA expression level measured by RT-PCR analysis. The log-rank test was used to identify relations between MFS (metastasis-free survival) and *PLK1* mRNA expression. Area under the curve analyses was performed to identify a cut-point to divide the cohort into two relevant PLK1 expression subgroups (low and high PLK1 expression) in the global population and in the different breast cancer sub-groups. Statistical significance was assessed by the log-rank (Mantel–Cox) test. Source data from **a**–**c** are provided as a Source Data file.

To assess whether the expression of PLK1 and the proliferation status of the PDX are correlated to volasertib response, we analysed the expression levels of *PLK1* and *Ki67* genes in the control xenografts of the seven PDX, by real-time polymerase chain reaction (RT-PCR) analysis. The expression of both *PLK1* and *Ki67* genes was higher in PDX responding with complete response or tumour regression as compared to PDX with no response or partial response to volasertib (Fig. 5a). Next, we analysed *Ki67* expression changes in volasertib-treated xenografts to determine whether inhibition of proliferation was associated to response. *Ki67* expression was decreased in six PDX models: three in the group of responding tumours and three in the group of PDX responding with stable disease or progressive disease (Fig. 5b).

Moreover, as response to PLK1 inhibitors is associated with mitotic arrest of treated tumours^8,14^, we performed a pharmacodynamics study with two responder (HBCx-124 and HBCx-137) and two resistant PDX (HBCx-118 and HBCx-131) to analyse phospho-histone H3 (P-HisH3) expression (a marker of mitosis) at baseline and 24 h after a single drug administration. Results show an increase of P-HisH3 staining up to 10% of tumour cells in the two responder PDX and in 3% of tumour cell in the HBCx-131 model (Fig. 5b). In summary, these results indicate that tumour response to volasertib is greater in highly proliferating tumours and is associated with post-treatment mitotic arrest.

### PLK1 expression is a strong predictor of a shorter metastasis free survival

In order to determine the prognostic significance of PLK1 expression pattern in human breast tumours, we analysed *PLK1* mRNA levels in a large series of 441 primary breast tumours from patients with known clinical/pathological status and long-term outcome (Supplementary Table 2). Results in the global population showed that metastasis-free survival (MFS) of patients with high *PLK1*-expressing tumours (5-year MFS 68.4%; 10-year MFS 58.5%; 15-year MFS 49.8%) was shorter than that of patients with low *PLK1*-expressing tumours (5-year MFS 88.7%; 10-year MFS 77.4%; 15-year MFS 74.4%) (*P* < 0.0001; Fig. 5c).

This prognostic significance of *PLK1* expression status in the global population was restricted to the hormonal receptor (HR)-positive (HR+HER2+ and HR+HER2−) sub-group (*P* < 0.0001), and was not observed in the triple negative sub-group, as well as in the HR-HER2+ sub-group.

The prognostic significance of parameters identified in univariate analysis and *PLK1* expression status persisted, except for Scarff Bloom Richardson (SBR) histopathological grade and progesterone receptor (PR) status, in the Cox multivariate regression analysis of MFS (Supplemental Table 3).

As PLK1 promotes cell proliferation of various cancers^15,16^, we tested possible correlation between *PLK1* and various genes involved in cell cycle signalling pathway, i.e., *AURKA*, *MK67* and *NEK2*, quantified by RT-PCR analysis in the same cohort (Supplementary Fig. 4). We observed high positive association between *PLK1* and these three genes (*r* = +0.828, *P* < 0.0001 for *AURKA*; *r* = +0.749, *P* < 0.0001 for *MKI67*; *r* = +0.782, *P* < 0.0001 for *NEK2;* Spearman rank correlation test).

### PLK1 does not interact with ER in vitro

**Fig. 6:**
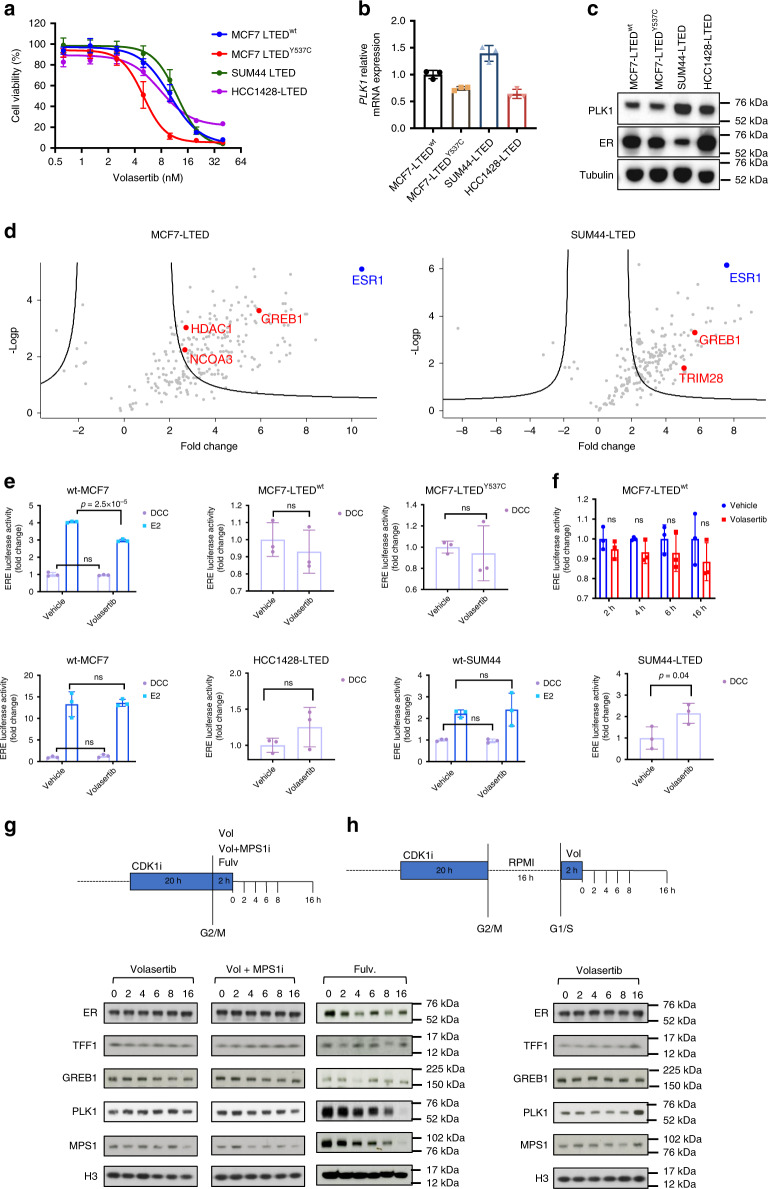
In vitro studies of volasertib and siRNA against PLK1. **a** Effect of escalating doses of volasertib on proliferation of MCF7-LTEDY537C, MCF7-LTEDwt, SUM44-LTED and HCC1428-LTED cell lines. Cell viability was analysed using a CellTiter-Glo assay and represented as percentage of vehicle control. Error bars represent mean ± SEM. *n* = 6 independent biological samples **b** Relative mRNA expression of PLK1 in MCF7-LTEDY537C, MCF7-LTEDwt, SUM44-LTED and HCC1428-LTED cell lines. Data assessed by RT-qPCR and represented as relative to MCF7-LTEDwt. *n* = 3 independent biological samples. Error bars represent means ± SD. **c** Immunobloting depicting changes in expression of ER and PLK1 in MCF7-LTEDY537C, MCF7-LTEDwt, SUM44-LTED and HCC1428-LTED cell lines. **d** Label-free quantitative analysis of ER interacting proteins in MCF7-LTEDY537C and SUM44-LTED cell lines. Volcano plot representing the logarithmic ratio of protein LFQ intensities in the RIME experiments plotted against negative logarithmic *p* values of the *t* test performed from triplicates (FDR threshold = 0.01, S0 = 2). ER and known interactors are highlighted in blue and red dots, respectively. **e** ER/ERE transactivation of wt-MCF7, MCF7-LTEDY537C, MCF7-LTEDwt, wt-SUM44, SUM44-LTED, wt-HCC1428 and HCC1428-LTED. Cells were transfected with an ERE-linked luciferase reporter and pCH110 (b-galactosidase control) and the following day treated with vehicle or volasertib (40 nM). Data expressed relative to vehicle control. Relative mRNA expression of oestrogen regulated genes TFF1, PGR and GREB1 after treatments with siControl or siPLK1 in several models of endocrine sensitivity. Expression assessed by RT-qPCR. *n* = 3 independent biological samples. Two-sided *t* test, no multiple comparison. Error bars represent mean ± SEM. **f** ER/ERE transactivation of MCF7-LTEDwt after transfection with an ERE-linked luciferase reporter and pCH110 (b-galactosidase control) and treatment with vehicle or volasertib (50 nM) for 2, 4, 6 and 16 h. Data expressed relative to vehicle control. *n* = 3 independent biological samples, interleaved scatter bars (mean ± SD) Two-sided *t* test. **g**, **h** Immunobloting assessing timecourse changes in protein abundance after release of 2 h treatment with volasertib (50 nM), fulvestrant (100 nM) or combination of volasertib and MPS1 inhibitor (150 nM), **g** without or **h** with medium synchronisation for 16 h. Source data are provided as a Source Data file.

Previous studies suggest PLK1 may influence ER transcriptional activity^10,11^. However, expression of ERGs was not decreased in volasertib-treated tumours of HBCx-137 PDX (Fig. 1g). In order to analyse PLK1 and ER interaction in vitro, a panel of cell lines resistant to long-term oestrogen-deprivation (LTED), which harboured naturally occurring ESR1^Y537S^, ESR1^Y537C^ or ESR1 wild-type (wt)^17^, were subjected to escalating concentrations of volasertib. In keeping with our PDX models, the drug caused a dose-dependent decreased in the proliferation of all the cell lines tested (Fig. 6a). MCF7-LTED^Y537C^ were the most sensitive with an IC_50_ of 5 nM compared with MCF7-LTED^wt^, HCC1428-LTED and SUM44-LTED which had IC_50_ values between 10 and 16 nM. Expression of PLK1 was not a determinant of sensitivity either at the RNA or protein level (Fig. 6b, c).

In order to assess the interaction of ER with PLK1, we carried out comparative RIME (rapid immunoprecipitation mass spectrometry of endogenous proteins) and dimethyl-labelling in wt-MCF7 and wt-SUM44 cells in the presence of oestrogen and MCF7-LTED and SUM44-LTED in the absence of oestrogen. (Fig. 6d, Supplementary Fig. 5a and Supplementary Data 5). As expected, the most abundant interaction in all models was with ER. Furthermore, the main ER-interactors were NCOA3, GREB1, TRIM28 and HDAC1. No interaction with PLK1 was evident in any of the models and interrogation of publicly available data similarly showed no interaction^18–20^. In order to test this further, targeted co-IPs were performed in MCF7-LTED cells confirming the lack of interaction (Supplementary Fig. 5b).

As ER-transcriptional activity may be affected by PLK1 indirectly^11^, we used siRNA targeting *PLK1* and treatment with volasertib to assess ER-mediated transactivation and the impact of PLK1 perturbation on the expression of specific oestrogen regulated genes (ERGs). Inhibition of PLK1 with volasertib had minimal or no impact on ER/ERE-mediated transactivation in wt-MCF7, wt-HCC1428 or wt-SUM44 in the presence or absence of oestrogen (Fig. 6e). Furthermore, no significant impact was evident in the LTED models with the exception of SUM44-LTED where volasertib caused a significant rise in ER/ERE reporter activity, an observation in contrast to previous studies^11^. In addition, siPLK1 caused no significant decrease in the expression of prototype ERGs (*TFF1*, *GREB1* and *PGR*) (Supplementary Fig. 6).

In order to address the potential impact of time on the ability of PLK1 to influence ER-mediated transactivation, we assessed the effect of volasertib treatment on ER/ERE-luciferase activity over 16 h in MCF7-LTED cells. No impact on hormone independent transcriptional activity was evident (Fig. 6f). In order to address the potential that PLK1 influences ER at different points during cell cycle, we synchronised MCF7-LTED cells using a CDK1 inhibitor (RO3306) followed by treatment with volasertib to block PLK1 activity or volasertib plus the MPS1 inhibitor (BOS172722). Fulvestrant, which down-regulates ER activity was used as a positive control (Fig. 6g, h). As expected, inhibition of PLK1 had no impact on the abundance of TFF1, GREB1 or ER. However, a time dependent decrease in MPS1 was evident in the presence of volasertib and BOS172722 indicative of cell cycle arrest. Contrastingly, fulvestrant caused a reduction in ER, TFF1 and GREB1 which appeared cyclical. Noteworthy, fulvestrant caused a reduction in MPS1 and PLK1, 4 h post-treatment, concordant with cell cycle arrest (Fig. 6g) and our previous observations.

**Fig. 7:**
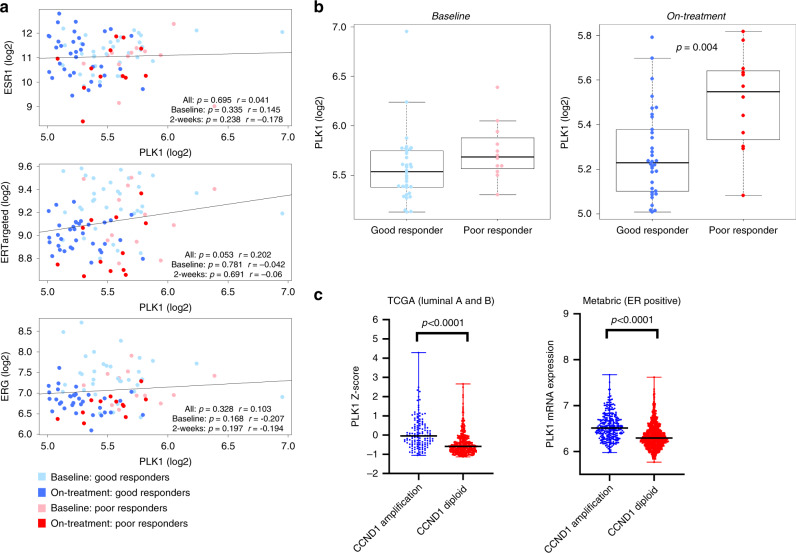
PLK1 correlation plots in clinical samples. **a** Correlation plots between PLK1 and ESR1, ERTargeted or ERG metagenes. Light blue dots—good responders at baseline; light red dots—poor responders at baseline; dark blue dots—good responders on-treatment (2 weeks); Dark red dots—poor responders on-treatment (2 weeks). Pearson correlation coefficients are shown. **b** Boxplot (box representing the median, first and third quartiles; whiskers representing min to max excluding outliers) highlighting the association of PLK1 expression with good and poor responders to anastrozole both at baseline and on-treatment. *p* Value < 0.05 by Student *t* test is shown. Light blue dots—good responders at baseline; light red dots—poor responders at baseline; dark blue dots—good responders on-treatment (2 weeks); dark red dots—poor responders on-treatment (2 weeks). **c** PLK1 expression in ER-positive tumours of TCGA and Metabric according to *CCND1* amplification. Data are presented as scatter dot plot with line indicating the median with range.

Finally, we assessed the association of PLK1 with expression of ERG in a cohort of 69 postmenopausal women with stage I to IIIB ER+ early BC who had received single agent neoadjuvant anastrozole^21^ (Fig. 7a). Global gene expression data from paired samples taken at baseline and 2-week post-treatment were available. *PLK1* expression did not correlate with ERGs (rho = 0.103, *p* = 0.328) or *ESR1* (rho = 0.041, *p* = 0.695) (Fig. 7a). Furthermore, correlations of PLK1 with individual ERGs similarly showed no association (*TFF1*, *PGR*, *GREB1*, *PDZK1*, *MYC* and *RET*) (Supplementary Fig. 7). Contrastingly, *PLK1* expression strongly correlated with cell cycle regulated genes including *CCND1* (Supplementary Fig. 8). On the same cohort of patients we found that high on-treatment expression of *PLK1* associates to a poor response to anastrozole (Fig. 7b). Finally, we looked at *PLK1* expression in *CCND1-*amplified as compared to *CCND1-*diploid tumours in the TCGA and Metabric breast cancer datasets (Fig. 7c). In both cohorts, *PLK1* expression was higher in the group of *CCND1*-amplified breast tumours.

In conclusion, these data suggest PLK1 does not influence ER-activity in either a ligand-dependent or -independent manner and that high on-treatment expression of PLK1 correlated with poor response to ET.

## Discussion

In this study we developed PDX from bone metastasis biopsies of patients progressing on ET to identify new therapeutic targets in a context of hormone-resistance. The transcriptomic analysis of bone metastasis-derived PDX as compared to patients’ early breast tumours, showed an enrichment in gene sets associated with G2/M checkpoint and mitosis. Molecular profiling on matched bone metastasis and primary tumours are limited due to the challenge of obtaining DNA and RNA from decalcified samples. However one study performed a RNA sequencing of 11 patient-matched primary BC and decalcified bone metastasis and reported G2/M checkpoint as one of the three most significantly enriched gene sets in bone metastases^22^. The same study found downregulation of EMT, NF-KB/TNF and different stem cell gene sets in bone metastases, that were also downregulated in our PDX. The striking similarity of our results with those reported by Priedigikeit et al. suggests that the up-regulation of mitotic genes in PDX is not an artefact of tumour engraftment but rather a characteristic of the bone metastasis. Furthermore, a RNAseq analysis of matched primary breast tumours and tamoxifen-treated liver metastases identified cell cycle and DNA replication as up-regulated pathways in metastases^23^.

At the genomic level, five out of seven PDX carried amplification or CN gains of *CCND1* gene with additional alterations in cell cycle genes *CCNE2*, *CDK7*, *AURKA* or *CCNB1*. For six patients, we could match the copy number profile of patient’s primary breast tumour and PDX (with the bone met. biopsy in two cases) and found a high concordance of CNA. Common oncogenic drivers such as *CCND1* and *FGFR1* or *FGFR2* were amplified in the primary breast tumours and can be considered early events in these patients’ tumours. Amplification of *CCND1* is one of the major genetic determinant of hyperactivity of the cyclin D–CDK4/6 pathway that occurs frequently in ER positive BC^24,25^. The CCND1–CDK4/6 axis promotes G1 exit through RB phosphorylation which results in activation of a proliferation transcriptional programme and phosphorylation cascades that drive cancer cells into the S phase, DNA replication and mitosis^26^.

If *CCND1* amplification was present in the primary tumours, *CDKN2A* homozygous deletion appeared in the bone metastases (HBCx-131) or in the PDX (HBCx-134), in patients who recurred 10 years and 1 year, respectively, after treatment with an aromatase inhibitor. *CDKN2A* loss, that promotes cell cycle progression by increasing CDK4/6 activity^27^, has been reported in therapy exposed BC metastases in different studies^22,28^, suggesting that this genetic event might contribute to endocrine resistance or tumour progression of metastatic BC.

Different targetable kinases associated to mitosis were up-regulated in bone metastases, including *PLK1*, *AURKA*, *AURKB*, *CDK1* and *MPS1*. Importantly, we found that four out of seven PDX models responded dramatically (with complete response or tumour regression) to the PLK1 inhibitor volasertib. The efficacy of PLK1 inhibitors in BC has been reported in triple-negative BCs^29^ or ER + BC cell line derived xenografts^11^. Our results show that PLK1 is a therapeutic target in PDX models of advanced ER-positive BC. Those models showing the greatest sensitivity to volasertib carried concomitant amplification or CN gains of *CCND1* and *CCNE2* both of which are involved in RB inactivation driving tumour cells through the S phase, as well as CN gains in *CCNB1* and *CDK7* (HBCx-139) or *AURKA* (HBCx-124), genes required for G2/M progression^26^. These data suggest that tumours driven by CN gain in different key regulatory components of the cell cycle might respond dramatically to PLK1 inhibition. Accordingly, the proliferation status of the PDX, measured by PLK1 and Ki67 expression analysis in baseline tumours, was higher in responder PDXs as compared to resistant.

Interestingly, *CCND1*-driven PDX with high response to volasertib harbour amplification or CN gain of *RSF1*, a marker of Integrative Cluster 2 in the BC integrative classification^30^. The Integrative Cluster 2 represents a particularly high-risk subgroup of luminal B BC patients, characterised by two separate amplicons in chromosome 11: one at 11q13.3 (*CCND1* amplification) and a separate peak from 11q13.5–11q14.1 centred around *PAK1*, *RSF1*, *C11orf67* and *INTS4*. Although our data are obtained from a small number of PDX models, they suggest that this group of patients could potentially benefit from PLK1 targeting.

Combination of volasertib with fulvestrant was less efficient than volasertib alone in two PDX. This could be explained by the decreased expression of *PLK1* in fulvestrant-treated tumours, consistent with published data showing that fulvestrant downregulates genes associated to cell proliferation and mitosis^31^. These results also suggests that the up-regulation of *PLK1* is not driving endocrine resistance in these tumours but is rather a consequence of an increased proliferation status of metastases. Furthermore, the expression of three ERGs (*TFF1*, *GREB1* and *PR*) was not decreased in treated tumours of a PDX highly responder to volasertib, indicating that *PLK1* inhibition and response to volasertib are not associated with an impaired ER-dependent transcription. These results are not in line with previous data reported in cell line models of endocrine resistance showing that PLK1 targeting with siRNA or volasertib inhibits ER-dependent transcription^10,11^. To further explore the role of PLK1 in ER-mediated transcription, we used different cell lines models of endocrine resistance and confirmed that PLK1 does not influence ER-activity in either a ligand-dependent or -independent manner.

In clinical practice, the combination of CDK4/6 inhibitors and ET is now standard of care for metastatic ER + BC. Our results show that the three tumours responding to volasertib are partially responsive to palbociclib and PDX with primary resistance to palbociclib were also resistant to volasertib, suggesting that cell cycle is the common determinant of response to CDK4/6 and PLK1 inhibitors. Moreover, our data show that PDX models driven by *AKT1*, *mTOR* or *NF1* mutations preferably respond to AKT1, mTOR and MEK inhibitors and this finding might have important clinical impact for the choice of first line treatment in this group of patients. Everolimus is approved for the treatment of metastatic BC^32^ and the AKT1 inhibitor Capivasertib has demonstrated anti-tumour activity in most patients with metastatic AKT1-mutant tumours enroled in the NCI-MATCH trial^33^.

In the clinic, acquired resistance to CDK4/6 inhibitors occurs frequently and represents the next major clinical challenge for the treatment of advanced ER + BC. Therefore, we established 2 PDX with acquired resistance to palbociclib, both of which conserved RB phosphorylation. The genomic analysis of palbociclib-resistant PDX did not reveal any change in CN level nor emergence of mutations in genes associated to cell cycle. Further analysis are ongoing to identify potential mechanisms of resistance in these PDX. The striking response to PLK1 inhibition in these PDX indicate that loss of G1/S dependency can be targeted with inhibitors of the G2/M cell cycle transition. In addition, we showed that targeting PLK1 might be a relevant therapeutic strategy in a context of secondary resistance to palbociclib and cross-resistance to PI3Kα inhibitor.

PLK1 inhibitors, including volasertib, have been shown to arrest tumour cells in mitosis, with an increase in the polyploid cell population that lead cancer cells to mitotic death^34^. Accordingly, the increase of histone H3 phosphorylation, which occurs specifically in mitosis, and of *CENPE* expression, a gene specifically expressed during the G2/M phase^35,36^, indicates an increase of mitotic cells in volasertib treated xenografts.

The RT-PCR analysis of treated xenografts also shows that *Ki67* expression was decreased by volasertib treatment in six out ten PDX models. However *Ki67* expression decrease was not always associated with tumour regression, suggesting that inhibition of cell proliferation *per se* can not be considered a marker of response.

Finally, we found that high *PLK1* expression was a strong predictor of worse survival in a large cohort of ER positive BC patients. This is consistent with previous data showing that PLK1 expression is strongly correlated with a lower MFS^37,38^. Moreover, the 5-gene earlyR score, that includes *PLK1*, predicts outcome of ER positive BC^39^ and different studies showed the prognostic value of proliferation signatures in early BC^40,41^. Importantly, our analysis and the data from Loddo et al. show that at least 50% of ER-positive BC express PLK1 at high levels, suggesting that the proportion of patients that would benefit from a PLK1 targeting agent would be significant^37^.

In a second cohort of early stage BC treated by anastrozole in the pre-surgical setting^42^, we found that on treatment expression of *PLK1* was associated to a poor response to anastrozole.

In summary our results strongly support the development of PLK1 inhibitors in patients with endocrine-resistant metastatic ER+ BC including patients with acquired resistance to the CDK4/6 inhibitor palbociclib. A highly Ki67 index could represent a valuable biomarker to identify patients who could benefit from PLK1 targeting.

## Methods

### Patient-derived xenografts (PDX) establishment

PDX models of ER+ metastatic BC were obtained by engrafting biopsies from spinal bone metastases of ER-positive BC patients progressing under ET and treated with vertebroplasty to restore biomechanical vertebral properties (stabilisation) and reduce back pain. The protocol was approved by the Institut Curie Hospital committee (CRI: Comité de Revue Institutionnel). Bone metastasis biopsies were engrafted with informed consent from the patient into the interscapular fat pad of female Swiss nude mice (Charles River Laboratories), which were maintained under specific pathogen-free conditions. Their care and housing were in accordance with institutional guidelines and the rules of the French Ethics Committee: CEEA-IC (Comité d’Ethique en matière d’expérimentation animale de l’Institut Curie, National registration number: #118). The project authorisation no. is 02163.02. The housing facility was kept at 22 °C (±2 °C) with a relative humidity of 30–70%. The light/dark cycle was 12 h light/12 h dark.

### In vivo efficacy studies

Volasertib (BI 6727), AZD4547 (FGFR1,2,3 inhibitor), AZD5343 (AKT1 inhibitor), Trametinib (MEK inhibitor), Alpelisib (BYL-719) and palbociclib (CDK4/6 inhibitor) were purchased from MedchemExpress. Volasertib was administered orally 4 days per week at a dose of 10 mg/kg. AZD4547, AZD5343, Trametinib, Alpelisib and palbociclib were administered orally 5 days per week at 12, 100, 1, 35 and 75 mg/kg, respectively. Everolimus (Certican, Novartis Pharma) was administered orally at a dose of 2.5 mg/kg 3 times per week. Fulvestrant (Faslodex, AstraZeneca, Macclesfield, UK) was administered by intramuscular injection at a dose of 50 mg/kg once a week.

For efficacy studies, tumour fragments were transplanted into female 8-week-old Swiss nude mice. Xenografts were randomly assigned to the different treatment groups when tumours reached a volume comprised between 100 and 200 mm^3^. Tumour size was measured with a manual calliper twice per week. Tumour volumes were calculated as *V* = *a* × *b*^2^/2, a being the largest diameter, *b* the smallest. Tumour volumes were then reported to the initial volume as relative tumour volume (RTV). Means (and SD) of RTV in the same treatment group were calculated, and growth curves were established as a function of time. For each tumour the percent change in volume was calculated as (*V*_f_ − *V*_0_/*V*_0_)/100, *V*_0_ being the initial volume (at the beginning of treatment) and *V*_f_ the final volume (at the end of treatment). A decrease in tumour volume of at least 50% was classified as regression, an increase in tumour volume of at least a 35% identified progressive disease and volumes changes between +35% and −50% were considered as stable disease^43^. The statistical analysis of tumour growth inhibition was performed with the Mann–Whitney test or the Dunn’s multiple comparisons test.

### Gene expression analysis

Gene expression microarrays were performed at the Genomics platform of Institut Curie. GeneChip Human 1.1 ST arrays were hybridised according to Affymetrix recommendations, using the Ambion WT Expression Kit protocol (Life Technologies) and Affymetrix labelling and hybridisation kits. Affymetrix CEL files were imported into the Gene Expression Workflow in Partek^®^ Genomics Suite version 7.0 (Partek Inc., St. Louis, MO, USA, www.partek.com). Background correction, quantile normalisation, log2 transformation, and probeset annotation were performed using default settings for the Robust Multichip Average (RMA) procedure.

Gene set enrichment analysis (GSEA, v4.0.3) software and MsigDB database (v7.0)^44^ were used to identify overrepresented biological functions for differentially expressed genes between PDX and primary breast tumours.

Enriched pathways were then represented as an interactive network, using EnrichmentMap^45^, a Cytoscape^46^ application that determine relationships between pathways. Pathways are represented by nodes and connected by edge if they shared common genes. Highly interconnected nodes are clustered in order to identify major biological processes. Affymetrix CEL files and normalised log2 RMA data are available at the GEO database (accession No. GSE146661; [https://www.ncbi.nlm.nih.gov/geo/query/acc.cgi?acc=GSE146661]).

### RNA extraction and RT-PCR analysis of PDX

RNA extraction was performed by using acid-phenol guanidium method^47,48^. Electrophoresis through agarose gel staining with ethidium bromide was performed to determine the RNA quality and 18S and 28S RNA bands were visualised under ultraviolet light. RNA was reverse transcribed in a final volume of 20 μl containing 1× RT buffer [500 mm each dNTP, 3 mm MgCl2, 75 mm KCl, and 50 mm Tris-HCl (pH 8.3)], 10 units of RNasinTM RNase inhibitor (Promega, Madison, WI), 10 mm DTT, 50 units of Superscript II RNase H-reverse transcriptase (Life Technologies, Inc., Gaithersburg, MD), 1.5 mm random hexamers (Pharmacia, Uppsala, Sweden), and 1 μg of total RNA. The samples were incubated at 20 °C for 10 min and 42 °C for 30 min, and reverse transcriptase was inactivated by heating at 99 °C for 5 min and cooling at 5 °C for 5 min.

We used protocols for PCR amplification described in detail elsewhere^49,50^. Briefly, we obtained quantitative values from the *C*_t_ value (cycle number) at which the increase in the fluorescence signal associated with exponential growth of PCR products was detected by the laser detector of the ABI Prism 7900 sequence detection system (Perkin-Elmer Applied Biosystems, Foster City, CA), using according to the manufacturer’s manuals (SDS Software v2.3).

The human TATA box-binding protein (TBP, GeBbank accession no. NM_003194) gene was used gene normalisation. Results, expressed as *N*-fold differences in target gene expression relative to the *TBP* gene and termed “Ntarget”, were calculated as Ntarget = 2Δ*C*_tsample_, where the Δ*C*_t_ value was determined by subtracting the average *C*_t_ value of target gene from the average TBP gene *C*_t_ value. The target gene values of the patients’ breast tumour samples were subsequently normalised such that the median of the target gene values for the ten normal breast tissues was 1.

The primer pairs used were: *TBP*: 5′-TGCACAGGAGCCAAGAGTGAA-3′ (upper) and 5′-CACATCACAGCTCCCCACCA-3′ (lower); *PLK1*: 5′-GCAGATCAACTTCTTCCAGGATCA-3′ (upper) and 5′- CGCTTCTCGTCGATGTAGGTCA-3′ (lower); *AURKA* 5′-GCATTTCAGGACCTGTTAAGGCTA-3′ (upper) and 5′- TGCTGAGTCACGAGAACACGTTT-3′ (lower); *MKI67* 5′-ATTGAACCTGCGGAAGAGCTGA-3′ (upper) and 5′- GGAGCGCAGGGATATTCCCTTA-3′ (lower); *NEK2* 5′-CCCTGTATTGAGTGAGCTGAAACTG -3′ (upper) and 5′- GCTCCTGTTCTTTCTGCTCCAAT-3′ (lower); *ESR1* 5′-CCACCAACCAGTGCACCATT-3′ (upper) and 5′- GGTCTTTTCGTATCCCACCTTTC-3′ (lower); PGR 5′-CGCGCTCTACCCTGCACTC-3′ (upper) and 5′-TGAATCCGGCCTCAGGTAGTT-3′ (lower); TFF1 5′-CATCGACGTCCCTCCAGAAGAG-3′ (upper) and 5′-CTCTGGGACTAATCACCGTGCTG-3′ (lower); *GREB1* 5′-CAAAGGGTGGTCTCCAGAATCTC-3′ (upper) and 5′-GACATGCCTGCGCTCTCATACT-3′ (lower); *CENPE* 5′-TGCCATACAAGGCTACAATGGTACT-3′ (upper) and 5′- ATGATCTTCTGAACCCATCATGGTA-3′ (lower); *PCNA* 5′-TCGATAAAGAGGAGGAAGCTGTTAC-3′ (upper) and 5′-GCAGACATACTGAGTGTCACCGTT-3′ (lower).

In the pharmacodynamics analysis, these *N*-fold differences values for each xenograft were normalised to yield a median value of 1 for the control xenograft group.

### Targeted NGS of PDX

Patient-derived xenografts genomes, with the exception of HBCx-180, were analysed by targeted NGS of 95 genes (Supplementary Data 1), chosen among the most frequently mutated genes in breast cancer (>1%)^7^. Targeted sequencing was employed for variant calling instead of exome sequencing in order to be more confident with the results since the depth is up to ten times higher than WES, allowing to detect variants even of low frequencies. Briefly, NGS was performed on an Illumina HiSeq2500 sequencer and the genomic variants were annotated with COSMIC and 1000 genome databases^51^. Reads were aligned using the Burrows–Wheeler Aligner (BWA) software, allowing up to 4% of mismatches with the reference. Variants with low allelic frequency (<5%) or low coverage (<100×) were excluded from the analysis.

The parental and palbo-resistant HBcx-134 and HBCx-124, as well as the HBCx-180 PDX, were also sequenced with a targeted NGS panel (called “DRAGON”) that has been recently developed in the genetics department of our Institute. It is composed of 571 genes of interest in oncology from diagnosis, prognosis and theranostics (Supplementary Data 1). NGS primers were selected based on their specificity on the human genome.

Sequencing for both panels were performed on an Illumina HiSeq2500 with a 500–1000X coverage. Reads were aligned using BWA allowing up to 4% of mismatches with the reference. Only reads with a mapping quality higher than 20 were used for variant calling, performed with Genome Analysis ToolKit (GATK, v3.5) Unified Genotyper and annotated with COSMIC and 1000 Genome databases^51^. Variants with low allelic frequency (<5%) or low coverage (<100x) and a high 1000 Genome frequency (>0.1%) were excluded from the analysis. Data are available at the European Genome-phenome Archive (EGA) under the No. EGAS00001004268 [https://ega-archive.org/studies/EGAS00001004268].

Deleterious genomic alterations were defined as follows: (i) for oncogenes, only mutations driving to gain of function were considered (i.e., hotspots missense mutations, in-frame insertions/deletions/splicing described as oncogenic), (ii) for tumour suppressor genes (TSG), only mutations driving to loss of function were considered (i.e., biallelic truncating alterations (nonsense mutations, frameshift insertions/deletions/splicing) or monoallelic truncating alterations associated with heterozygous deletion detected by copy number analysis).

### Whole-exome sequencing

DNA from 6 PDX (HBCx-118, HBCx-124, HBCx-131, HBCx-134, HBCx-137 and HBCx-142) with the matched patient’s primary breast tumours and/or matched bone metastasis were extracted and whole-exome libraries were prepared using SureSelect Human Clinical Research Exome Regions kit (Agilent). All patients signed a specific consent for tumour and normal DNA sequencing. Sequencing was done on Illumina NovaSeq 6000 system, generating 100 × 100 bp paired-end reads. Enriched tumour and germline DNAs were sequenced with an average depth of ~100× and ~30× uniquely mapped reads, respectively. For all samples, sequenced reads were aligned to the hg19 human reference genome using Bowtie2 (v2.1.0)^52^. Only alignments intersecting the targeted sequence were conserved. Duplicate reads were identified and discarded using Picard Tools (v1.130, https//broadinstitute.github.io/picard/). Finally, local realignment around small insertions and deletions (indels) and base quality recalibration were performed using GATK (v3.5)^53^. For tumour xenograft sequence data, a computational deconvolution of mouse and human reads was performed using XenofilteR R package (0.99.0)^54^.

Whole exome sequencing data are available at the European Genome-phenome Archive (EGA) (No. EGAS00001004268) [https://ega-archive.org/studies/EGAS00001004268].

### Copy number variant detection

Copy number was called from WES data using the FACETS (v0.5.2) R package^55^. Briefly, tumour samples and normal counterparts read counts were used as input to FACETS, which performs a joint segmentation of total- and allele-specific CNAs, and integer copy number calls corrected for tumour purity, ploidy and clonal heterogeneity. Segmented Log2 ratio from FACETS were then used as input for ABSOLUTE (v1.0.6)^56^ to determine modal copy number and cancer cell fractions (CCFs). Segments of 10 Mb or less with modal copy number greater than threefold the average ploidy were considered amplifications. Segments with modal copy number less than average ploidy-1 were defined as losses, and modal copy numbers of 0 were considered homozygous deletions.

### Affymetrix cytoscan HD array

Patients corresponding to HBCx-139, HBCx-176 and HBCx-180 did not provide the specific consent to perform WES on their normal and tumour samples. In absence of this consent, we performed cytoscan HD arrays to have copy number data from these PDX. Tumour DNA were profiled using Affymetrix Cytoscan HD array according to the manufacturer’s instructions^7^. Raw data were processed using the GAP methodology to obtain absolute copy number profiles^57^. CytoScan raw data are available in the GEO database (GSE149038) [https://www.ncbi.nlm.nih.gov/geo/query/acc.cgi?acc=GSE149038].

### Immunohistochemistry

For immunohistochemistry studies, 3 μm-thick tissue sections from formalin-fixed paraffin-embedded tissue were de-paraffinized, rehydrated and unmasked in target retrieval solution pH 9 for 15′ at 95 °C.

Anti-PLK1 (208G4) was provided by Cell Signalling, the detection was performed by immunoperoxidase technique and 3,3′-Diaminobenzidine tetrahydrochloride hydrate (DAB) chromogenic substrate revelation on the BOND RX using the Bond Polymer Refine Detection Kit (Leica) according to the manufacturer’sprotocols. Following blocking of endogenous peroxidase activity and inhibition of non-specific staining, the diluted antibody (1/50) was applied to slides for 60 min at room temperature. After washing with PBS the slides were incubated with polymeric horseradish peroxidase (HRP)-linker antibody conjugate system for 8 min. A DAB substrate solution was used to detect immunoreactive signals. The sections were counterstained with Mayer’s Hematoxylin.

Anti Phospho-Rb (Ser807/811) (D20B12) and anti FGFR1 (D8E4) were purchased from Cell Signalling (Rabbit Monoclonal #8516 and #9740) and respectively used at 1:400 and 1:200, after unmasking in citrate buffer pH6 for anti-Phopsho-Rb and in EDTA buffer pH8 for Anti FGFR1. Phospho-p44/42 MAPK (Erk1/2) (Thr202/Tyr204) (D13.14.4E) rabbit monoclonal antibody (Cell Signalling #437) was used at 1:400. Phospho-histone H3 (SER10) rabbit polyoclonal antibody (Cell Signalling #9701) was used at 1:100. Ki67 monoclonal antibody (clone SP6, TermoFisher # RM9106S1) was used at 1:100. All steps were performed in the Discovery XT autostainer (VENTANA Medical Systems, Roche) using an Omni-UltraMap HRP XT Kit according to the manufacturer protocol.

A semi-quantitative histologic score [*H*-score: intensity (0–3) × frequency (0–100%)] was performed (score 0: negative staining, score 1: weak staining,score 2: moderate staining, score 3: strong staining). Ki67 was scored as the percentage of nuclei-stained cells out of all cancer cells in the invasive front of the tumour regardless of the intensity in 10 × 400 high-power fields, 500–1000 tumour cells were counted in each case.

Immunohistochemically phospho-HisH3 stained slides were photographed with medium magnification using an Imager Z1 zeiss Microscope and a CamHRM Zeiss Camera and analysed in a blinded manner. Homogenous nuclear staining and nuclei with at least four or more stained foci were considered positive for expression. Positive- and negative-nuclear staining were quantified manually using four fields of approximatively 1000 cells for each tumour, and the percentage of P-His3 staining was evaluated in each fields.

### Cell culture

Human BC cell lines were obtained from the ATCC and Asterand, banked in aliquots to reduce phenotypic drift and identity confirmed using short tandem repeat analysis. Cells were also routinely screened for mycoplasma contamination. Wild-type cells were cultured in phenol red-free RPMI1640 containing 10% foetal bovine serum (FBS) and 1 nM estradiol (E2) and their respective LTED, modelling relapse on an aromatase inhibitor, were generated as reported previously^58^ and maintained in phenol red-free RPMI1640 containing 10% charcoal-dextran stripped FBS (DCC). All cell lines were stripped of steroids for 48–72 h prior to each experiment.

### qRT-PCR analysis of cell lines

RNA was extracted using the RNeasy kit (Qiagen), quantified and reverse-transcribed with SuperScriptIII First Strand Synthesis System (Invitrogen). Taqman gene expression assays (Applied Biosystems) were used to quantify *TFF1* (Hs00907239_m1), *GREB1* (Hs00536409_m1), CCNB1 (Hs99999188_m1), PLK1 (Hs00983277_m1), *ESR1* (Hs00174860_m1) and the house-keeping genes *FKBP15* (Hs00391480_m1). The relative quantity was determined using ΔΔ*C*_t_, according to the manufacturer’s instructions (Applied Biosystems).

### Transcriptional assays

Cell lines were seeded in 24-well plates in 10% DCC medium and left to acclimatise overnight. Transfection was performed with Fugene 6 at a ratio of 6:1 (Promega) with 0.1 µg of oestrogen response element linked luciferase (EREIItkluc) and 0.1 µg of β-galactosidase (pCH110) reporter constructs, as previously detailed^59^.

Twenty-four after, cells were treated with the drugs combinations and left for 24 h. A luminometer was used to measure Luciferase (Promega) and β-galactosidase (GalactoStar, Applied Biosystems) activities. Each experiment was performed three times with three to four replicates per treatment.

### Immunoblotting

Whole cell extracts were generated from cells cultured under basal conditions or after the treatments specified. Equal amounts of protein resolved by sodium dodecyl sulfate polyacrylamide gel electrophoresis (SDS-PAGE) and subjected to immunoblot analysis. Antigen–antibody interactions were detected with ECL-reagent (Amersham, UK) using the following antibodies: total-ER (Santa Cruz Biotechnology: sc-8002, clone F-10, 1:200), TFF1 (AbCam: ab92377, clone EPR3972 1:2000), GREB1 (ab72999, polyclonal, 1:1000), MPS11 (Millipore: 05-682 clone 3-472-1; 1:1000), H3 (abCam: Ab1791; 1:1000), PLK1 (Millipore: 05-044; clone 35-206; 1:1000) and tubulin (Sigma-Aldrich: T-9026, clone DM1A, 1:10000).

### Immunoprecipitation

Cell lysates were pre-cleared, incubated in primary antibodies IgG (Dako); ER (Santa Cruz Biotechnology: sc-543); PLK1 (Millipore: 05-044) at 4 °C overnight. Immuno-Complexes were recovered using protein G, washed six times in extraction buffer and resolved by SDS-PAGE, as specified above.

### RIME and dimethyl-labelling

RIME and stable isotope dimethyl-labelling were performed, as previously described^17,60,61^. Briefly, cells were labelled with the medium and light isotope reagent. Labelled samples were pooled at an approximate 1:1 ratio, dried down and fractionated using 12 cm IPG strip pH 3-10^62^. RIME and dimethyl-label fractions were desalted (SUM SS18V, The Nest Group Inc) and run through LC-MS/MS using LTQ Velos Orbitrap MS. Raw data for RIME and dimethyl-labelling were analysed using MaxQuant 1.5.1.0^62,63^. Statistical analysis was performed using Perseus software (version 1.6.1.3)^64^. Raw data have been deposited to the ProteomeXchange Consortium via PRIDE partner repository with the dataset identifier PXD004648 (MCF-7) and PXD004807 (SUM44).

### Institut Curie clinical cohort

Samples of 441 invasive primary breast cancers excised from patients treated at Institut Curie—Hôpital René Huguenin have been analysed^65^. All patients cared in our institution before 2007 were informed that their tumour samples might be used for scientific purposes and had the opportunity to decline. After 2007, patients gave their approval by signed inform consent. This study protocol was approved by the local ethical committee (Breast Group of René Huguenin Hospital). Samples were istored in liquid nitrogen until RNA extraction. Tumour samples were included in the study with a proportion of tumour cells of at least 70%. All patients (mean age 61.8 years, range: 31–91 years) met the following criteria: primary unilateral nonmetastatic breast carcinoma with clinical, histological and biological data were available; no radiotherapy or chemotherapy before surgery; and full follow-up at Institut Curie—Hospital René Huguenin.

Modified radical mastectomy was performed in 275 cases (63.5%) and breast-conserving surgery plus locoregional radiotherapy in 158 cases (36.5%). The patients had a physical examination and routine chest radiotherapy every 3 months for 2 years, then annually. Adjuvant chemotherapy was administered to 86 patients, hormone therapy was administered to 170 patients and both treatments to 101 patients. The histological classification and the number of positive axillary lymph-nodes were determined at surgery. The SBR histoprognostic system was used to score the malignancy of infiltrating carcinomas.

The status of oestrogen receptor alpha (ERα); PR and human epidermal growth factor receptor 2 (ERBB2) was determined at the protein level by using biochemical methods (dextran-coated charcoal method, enzyme immunoassay or immunohistochemistry) and confirmed by real-time quantitative RT-PCR assays^66^.

The population was divided into four groups according to HR (ERα and PR) and HER2 status, as follows: two luminal subtypes [HR+/HER2+ (*n* = 50)] and [HR+/HER2− (*n* = 283)]; an HER2+subtype [HR−/HER2+ (*n* = 41] and a triple-negative subtype [HR−/HER2− (*n* = 67)]. Standard prognostic factors of this tumour set are presented in Supplemental Table 2. During a median follow-up of 9.1 years (range 4.3 months to 33.2 years), 173 patients developed metastasis. Ten samples of adjacent normal breast tissue from breast cancer patients and normal breast tissue from women undergoing cosmetic breast surgery were used as sources of normal RNA.

### Gene expression microarray analysis of breast tumours

Global gene expression and Ki67 data were available from core-cut biopsies belonging to 69 postmenopausal women with paired baseline and 2-week post-treatment of single agent neoadjuvant anastrozole. Detailed methodology for this work and patient demographics are published elsewhere^21^ (https://www.ncbi.nlm.nih.gov/geo/query/acc.cgi?acc=GSE153470). The Pearson correlation method was used to analyse correlation between *PLK1* and the other genes. Data were extracted, transformed, normalised and filtered using the same methods as performed for the cell lines^67^. This study received approval from an institutional review board at each site and was conducted in accordance with the 1964 Declaration of Helsinki and International Conference on Harmonisation/Good Clinical Practice guidelines. Written informed consent was obtained from each patient before participation.

### Statistical analysis

Median values and ranges of mRNA levels were used to analyse the distributions of target gene levels.

Different nonparametric tests, namely the chi-square test (relation between two qualitative parameters), the Mann–Whitney *U* test (relation between one qualitative parameter and one quantitative parameter) and the Spearman rank correlation test (relation between two quantitative parameters) were used to analyse the relationship between different target genes and between mRNA levels and clinical parameters. Differences were considered significant at confidence levels greater than 95% (*p* < 0.05).

To analyse the efficacy of PLK1 level to discriminate two populations (patients that developed/did not develop metastases) in the absence of an arbitrary cut-off value, data were summarised in an ROC (receiver operating characteristic) curve^68^. The AUC (area under curve) was calculated as a single measure for discriminate efficacy. MFS was determined as the interval between initial diagnosis and detection of the first metastasis. Survival distributions were estimated by the Kaplan–Meier method, and the significance of differences between survival rates were determined with the log-rank test. The Cox proportional hazards regression model was used to assess prognostic significance and the results are presented as hazard ratios and 95% confidence intervals.

## Supplementary information

Supplementary Information

Description of Additional Supplementary Files

Supplementary Data 1

Dataset2

Dataset3

Dataset4

Dataset5

Source Data file

## Data Availability

Affymetrix raw data files and normalised log2 RMA data are available at the Gene Expression Omnibus (GEO) database (accession No. GSE146661; [https://www.ncbi.nlm.nih.gov/geo/query/acc.cgi?acc=GSE146661]). Data from targeted NGS panels are available at the European Genome-phenome Archive (EGA) under the No. EGAS00001004268 [https://ega-archive.org/studies/EGAS00001004268]. Whole-exome sequencing data are available at the European Genome-phenome Archive (EGA) (No. EGAS00001004268) [https://ega-archive.org/studies/EGAS00001004268]. The source data for Figs. 1c, g; 5a–c; 6a–h, Supplementary Figs. 5b and 6 are available as a Source Data file. All other data that support the findings of this study are available from the corresponding author upon reasonable requests.

## Code availability

The GAP methodology was previously described in detail^57^. An implementation of the GAP pattern recognition and detection of copy numbers and genotypes based on segmented profiles is available at the GAP download site of Institut Curie Bioinformatics: http://bioinfo-out.curie.fr/projects/snp_gap/.
