# Supplementary material for: PLK1 inhibition exhibits strong anti-tumoral activity in CCND1-driven breast cancer metastases with acquired palbociclib resistance
Source: Nat Commun. 2020 Aug 13;11:4053. doi: 10.1038/s41467-020-17697-1 (PMC7426966; doi:10.1038/s41467-020-17697-1)
Supplement: Supplementary file 1 — Supplementary Information [file 41467_2020_17697_MOESM1_ESM.pdf]

Supplementary Figures

Supplementary Figure 1

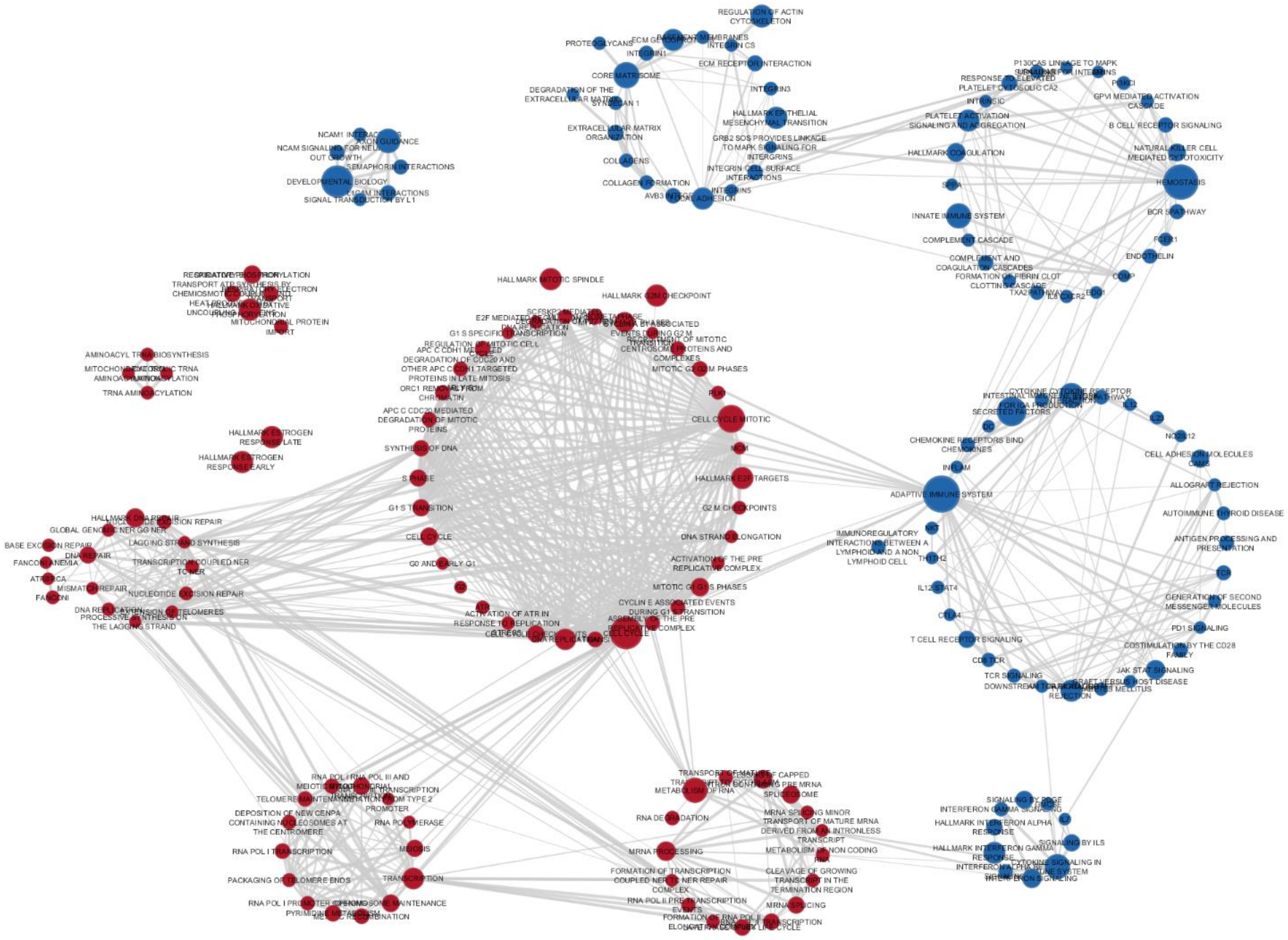

Supplementary Fig. 1 Integrative network analysis of enriched pathways in PDX samples as identified using *GSEA*. Upregulated and downregulated pathways are represented in red and blue, respectively.

Supplementary Figure 2

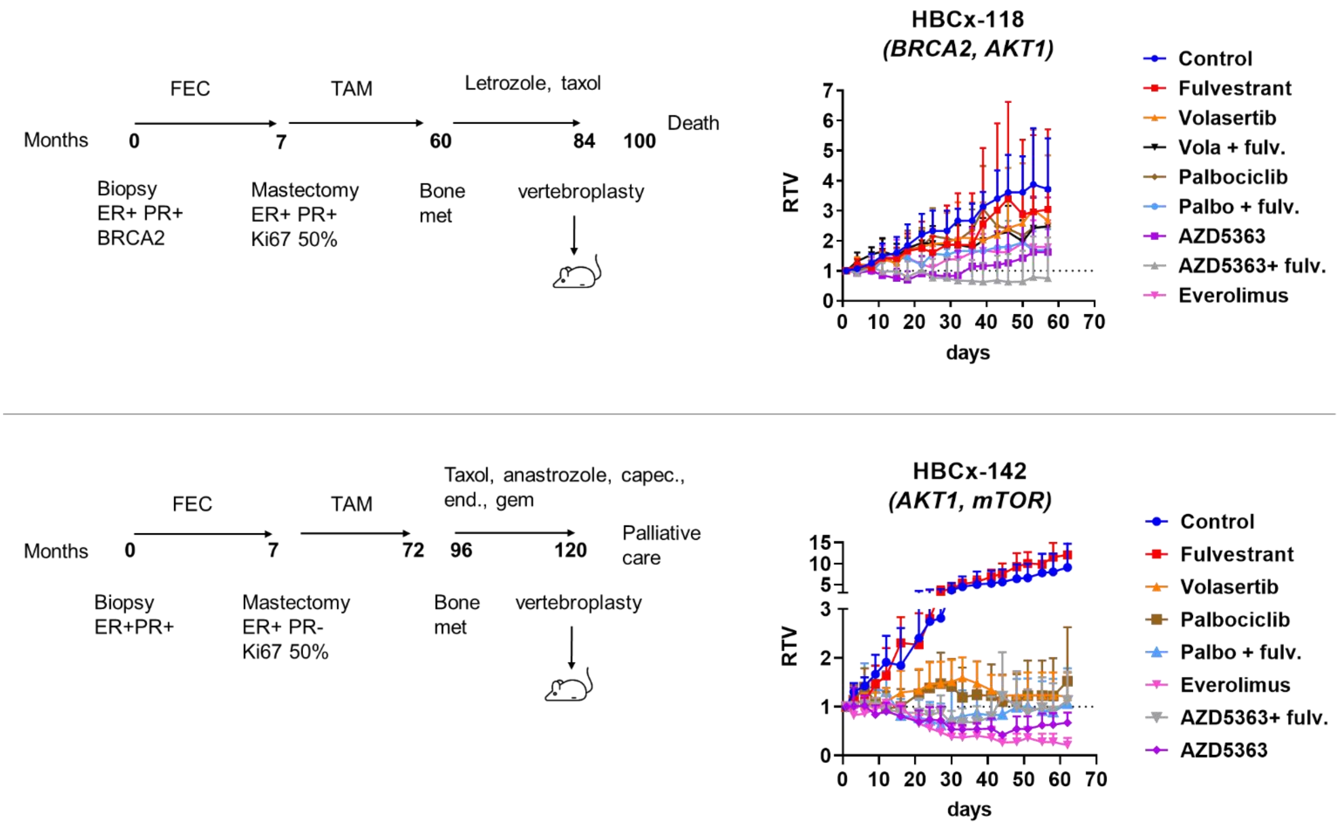

Supplementary Fig. 2 clinical histories and drug responses of PDX HBCx-118 and HBCx-142. Abbreviations: capec: capecitabine, end.: endoxan (cyclophosphamide), gem: gemcitabine. FEC: 5-Fu, epirubicin, cyclophosphamide. TAM: tamoxifen. Mean +/- SD. HBCx-118: n=4 for control, fulvestrant, volasertib, palbociclib, n=5 for fulv.+ volasertib, palbo+ fulv., n=7 for AZD5363, AZD5363+ fulv., n=6 for everolimus. HBCx-142: n=4 for control and everolimus groups, n=5 for the other groups.

Supplementary Figure 3

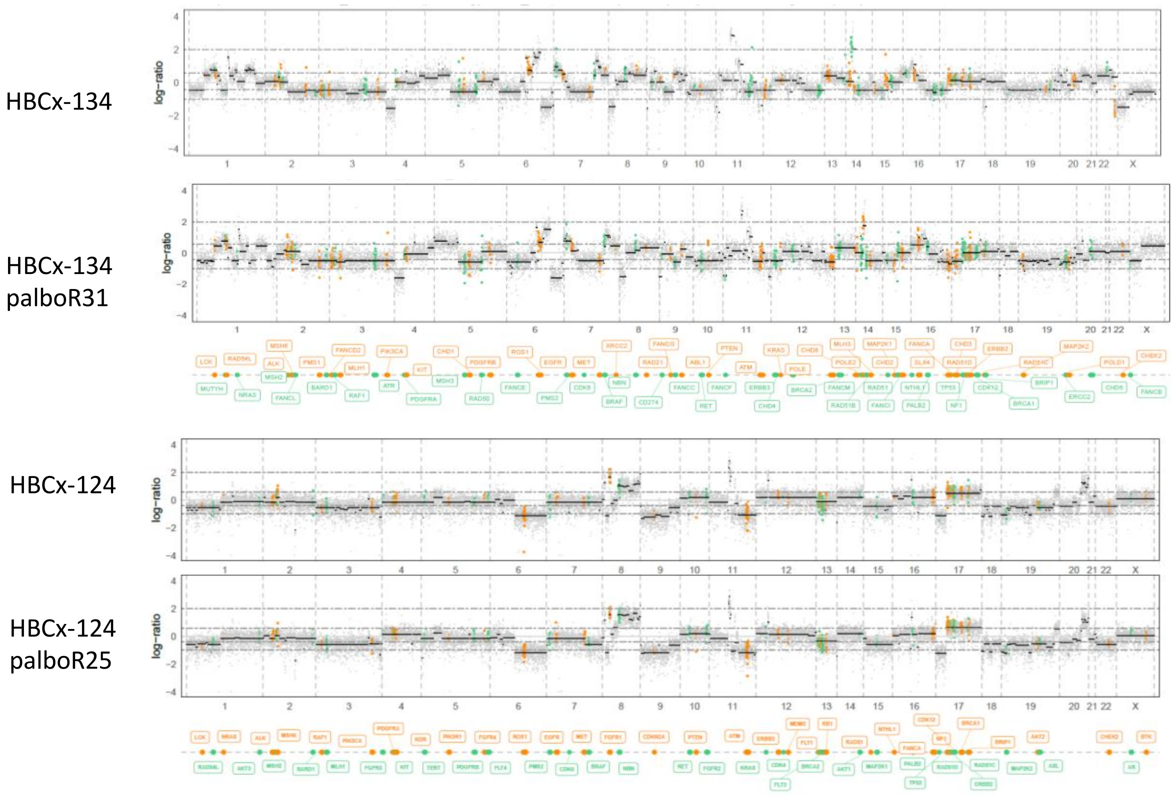

Supplementary Fig. 3 copy number alteration profiles of HBCx134 and HBCx-124 parental and palbociclib-resistant xenografts

Supplementary Figure 4

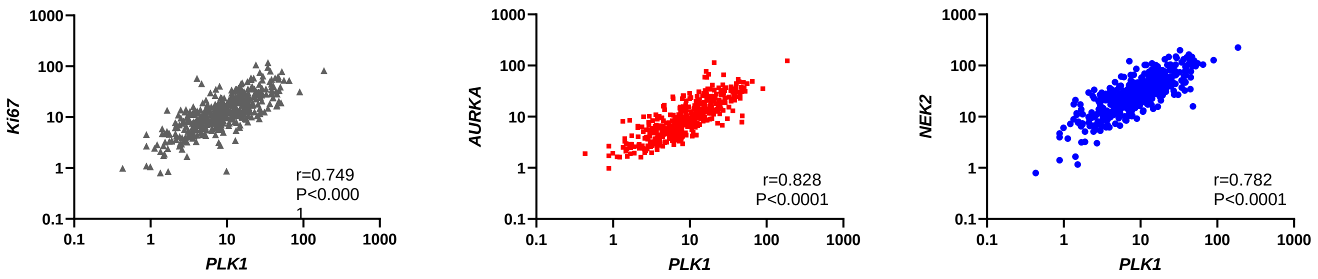

Supplementary Figure 4: correlation plots between *PLK1* gene and *Ki67, AURKA* and *NEK2* genes in the cohort of 441 breast cancers. Expression of *PLK1, Ki67, AURKA* and *NEK2* genes was determined by RT-PCR analysis (Spearman correlation analysis, p values are two-tailed).

Supplementary Figure 5

a

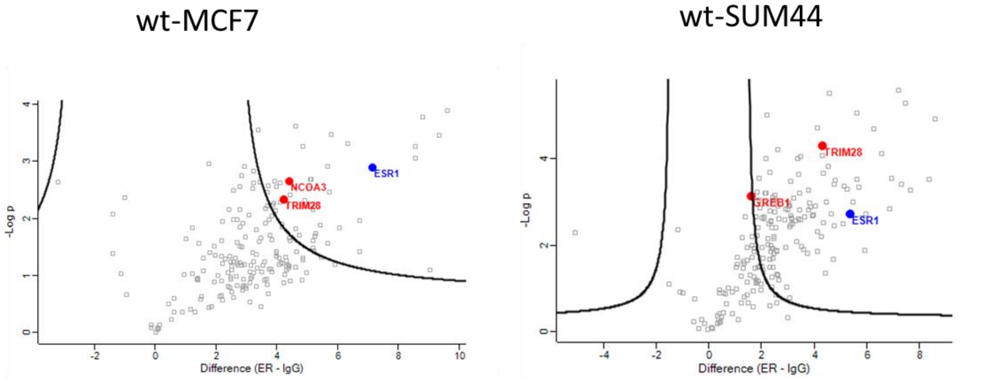

b

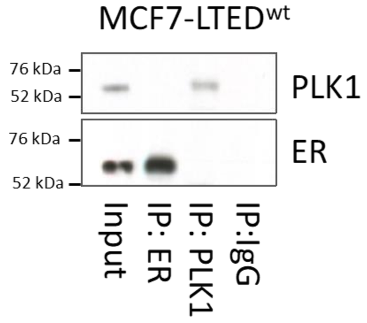

Supplementary Fig. 5 a, Label-free quantitative analysis of ER interacting proteins in wt-MCF7 and wt-SUM44 cell lines. Volcano plot representing the logarithmic ratio of protein LFQ intensities in the RIME experiments plotted against negative logarithmic p-values of the t test performed from triplicates (FDR threshold = 0.01, S0 = 2). ER and known interactors are highlighted in blue and red dots, respectively. **b,** MCF7-LTED^{wt} cells were immunoprecipitated for IgG, ER or PLK1 and immunoblotted ER and PLK1.

Supplementary Figure 6

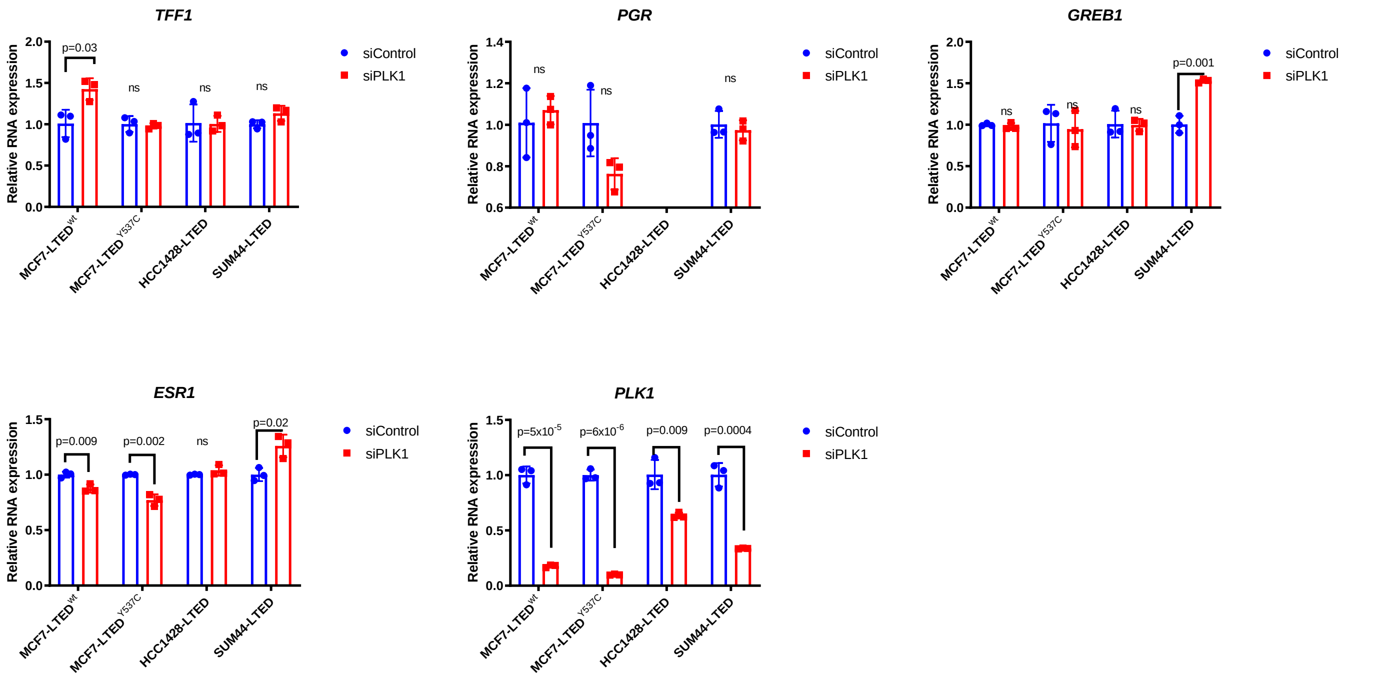

Supplementary Fig. 6 Relative mRNA expression of estrogen regulated genes *TFF1*, *PGR*, *GREB1* together with *ESR1* after treatments with *siControl* or *siPLK1* in several models of endocrine resistance. Expression assessed by RT-qPCR. Data represented relative to MCF7-LTED^{wt}. Interleaved scatter with bar (mean ± SD). n= 3 independent biological samples

Supplementary Figure 7

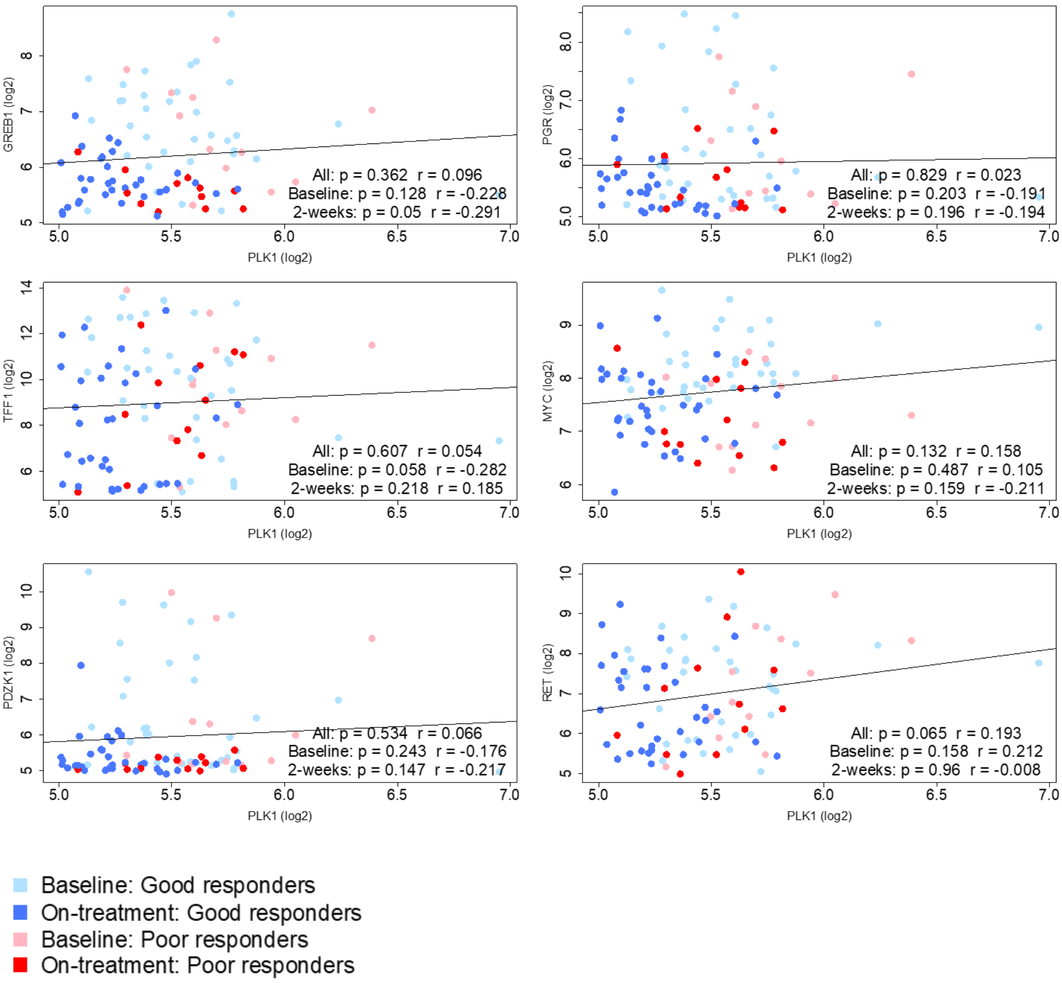

Supplementary Fig. 7 Correlation plots between *PLK1* and *TFF1*, *PGR*, *GREB1*, *PDZK1*, *MYC* and *RET*. Light blue dots- good responders at baseline; Light red dots- poor responders at baseline; Dark blue dots- good responders on-treatment (2-weeks); Dark red dots- poor responders on-treatment (2-weeks). Pearson correlation coefficients.

Supplementary Figure 8

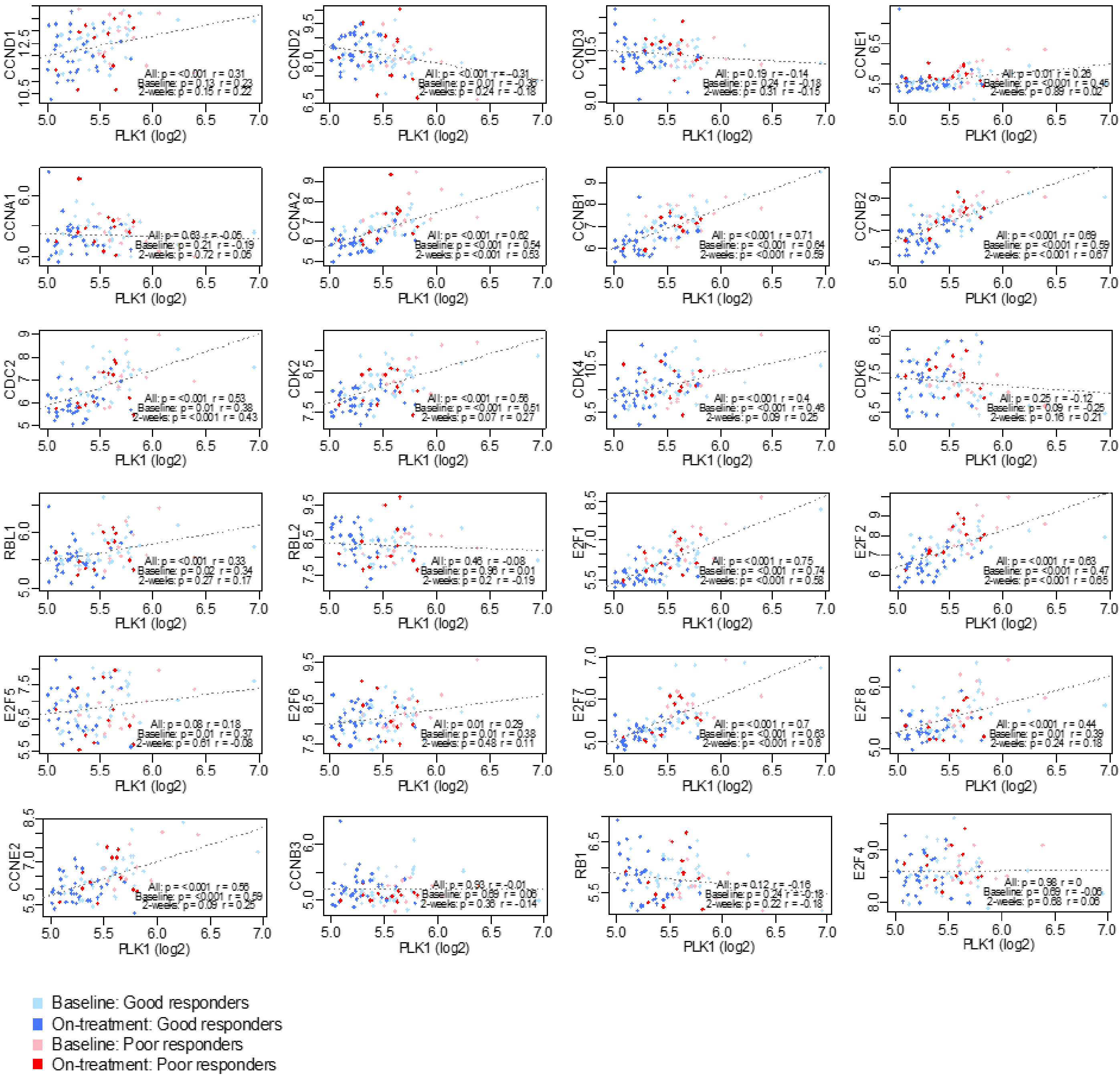

Supplementary Fig. 8 Correlation plots between *PLK1* and several cell cycle regulating genes. Light blue dots- good responders at baseline; Light red dots- poor responders at baseline; Dark blue dots- good responders on-treatment (2-weeks); Dark red dots- poor responders on-treatment (2-weeks). Pearson correlation coefficients.

Supplementary tables

Supplementary Table 1: GSEA (Gene Set Enrichment Analysis) of gene expression arrays in PDX models as compared to patients' primary breast tumours.

**GSEA report for PDX as compared to primary tumors**							
NAME	GS<br>follow link to MSigDB	SIZE	ES	NES	NOM p-va	FDR q-val	FWER p-val
HALLMARK_E2F_TARGETS	HALLMARK_E2F_TARGETS	198	0.656	3.153	0.000	0.000	0.000
HALLMARK_G2M_CHECKPOINT	HALLMARK_G2M_CHECKPOINT	199	0.590	2.791	0.000	0.000	0.000
HALLMARK_MYC_TARGETS_V1	HALLMARK_MYC_TARGETS_V1	198	0.490	2.324	0.000	0.000	0.000
HALLMARK_MYC_TARGETS_V2	HALLMARK_MYC_TARGETS_V2	56	0.575	2.240	0.000	0.000	0.000
HALLMARK_OXIDATIVE_PHOSPHORYLATION	HALLMARK_OXIDATIVE_PHOSPHORYLATION	198	0.440	2.084	0.000	0.000	0.000
HALLMARK_DNA_REPAIR	HALLMARK_DNA_REPAIR	141	0.479	2.075	0.000	0.000	0.000
HALLMARK_ESTROGEN_RESPONSE_EARLY	HALLMARK_ESTROGEN_RESPONSE_EARLY	198	0.403	1.889	0.000	0.000	0.001
HALLMARK_ESTROGEN_RESPONSE_LATE	HALLMARK_ESTROGEN_RESPONSE_LATE	198	0.388	1.833	0.000	0.001	0.003
HALLMARK_MITOTIC_SPINDLE	HALLMARK_MITOTIC_SPINDLE	198	0.379	1.766	0.000	0.001	0.007
HALLMARK_SPERMATOGENESIS	HALLMARK_SPERMATOGENESIS	133	0.371	1.653	0.000	0.003	0.017
HALLMARK_MTORC1_SIGNALING	HALLMARK_MTORC1_SIGNALING	197	0.342	1.598	0.000	0.005	0.034
HALLMARK_UNFOLDED_PROTEIN_RESPONSE	HALLMARK_UNFOLDED_PROTEIN_RESPONSE	111	0.352	1.520	0.000	0.010	0.080
HALLMARK_GLYCOLYSIS	HALLMARK_GLYCOLYSIS	198	0.260	1.203	0.089	0.119	0.642
HALLMARK_PI3K_AKT_MTOR_SIGNALING	HALLMARK_PI3K_AKT_MTOR_SIGNALING	105	0.229	0.989	0.480	0.484	0.987
**GSEA report for primary tumors as compared to PDX**							
NAME	GS<br>follow link to MSigDB	SIZE	ES	NES	NOM p-va	FDR q-val	FWER p-val
HALLMARK_EPITHELIAL_MESENCHYMAL_TRANSITION	HALLMARK_EPITHELIAL_MESENCHYMAL_TRANSIT	197	-0.835	-3.074	0.000	0.000	0.000
HALLMARK_ALLOGRAFT_REJECTION	HALLMARK_ALLOGRAFT_REJECTION	194	-0.757	-2.765	0.000	0.000	0.000
HALLMARK_INFLAMMATORY_RESPONSE	HALLMARK_INFLAMMATORY_RESPONSE	197	-0.733	-2.741	0.000	0.000	0.000
HALLMARK_COMPLEMENT	HALLMARK_COMPLEMENT	195	-0.732	-2.692	0.000	0.000	0.000
HALLMARK_KRAS_SIGNALING_UP	HALLMARK_KRAS_SIGNALING_UP	193	-0.726	-2.685	0.000	0.000	0.000
HALLMARK_COAGULATION	HALLMARK_COAGULATION	135	-0.743	-2.678	0.000	0.000	0.000
HALLMARK_TNFA_SIGNALING_VIA_NFKB	HALLMARK_TNFA_SIGNALING_VIA_NFKB	197	-0.713	-2.676	0.000	0.000	0.000
HALLMARK_INTERFERON_GAMMA_RESPONSE	HALLMARK_INTERFERON_GAMMA_RESPONSE	192	-0.716	-2.641	0.000	0.000	0.000
HALLMARK_IL6_JAK_STAT3_SIGNALING	HALLMARK_IL6_JAK_STAT3_SIGNALING	87	-0.740	-2.507	0.000	0.000	0.000
HALLMARK_IL2_STAT5_SIGNALING	HALLMARK_IL2_STAT5_SIGNALING	198	-0.644	-2.396	0.000	0.000	0.000
HALLMARK_ANGIOGENESIS	HALLMARK_ANGIOGENESIS	36	-0.838	-2.359	0.000	0.000	0.000
HALLMARK_APOPTOSIS	HALLMARK_APOPTOSIS	158	-0.635	-2.323	0.000	0.000	0.000
HALLMARK_UV_RESPONSE_DN	HALLMARK_UV_RESPONSE_DN	142	-0.645	-2.320	0.000	0.000	0.000
HALLMARK_INTERFERON_ALPHA_RESPONSE	HALLMARK_INTERFERON_ALPHA_RESPONSE	92	-0.640	-2.191	0.000	0.000	0.000
HALLMARK_APICAL_JUNCTION	HALLMARK_APICAL_JUNCTION	199	-0.576	-2.151	0.000	0.000	0.000
HALLMARK_MYOGENESIS	HALLMARK_MYOGENESIS	200	-0.556	-2.062	0.000	0.000	0.000
HALLMARK_HYPOXIA	HALLMARK_HYPOXIA	196	-0.515	-1.918	0.000	0.000	0.000
HALLMARK_APICAL_SURFACE	HALLMARK_APICAL_SURFACE	44	-0.569	-1.716	0.009	0.002	0.038
HALLMARK_TGF_BETA_SIGNALING	HALLMARK_TGF_BETA_SIGNALING	54	-0.529	-1.659	0.007	0.003	0.071
HALLMARK_HEDGEHOG_SIGNALING	HALLMARK_HEDGEHOG_SIGNALING	35	-0.557	-1.633	0.011	0.005	0.102
HALLMARK_ADIPOGENESIS	HALLMARK_ADIPOGENESIS	194	-0.424	-1.577	0.001	0.008	0.182
HALLMARK_NOTCH_SIGNALING	HALLMARK_NOTCH_SIGNALING	32	-0.542	-1.535	0.030	0.012	0.272
HALLMARK_ANDROGEN_RESPONSE	HALLMARK_ANDROGEN_RESPONSE	99	-0.435	-1.510	0.014	0.015	0.342
HALLMARK_XENOBIOTIC_METABOLISM	HALLMARK_XENOBIOTIC_METABOLISM	199	-0.400	-1.486	0.007	0.020	0.432
HALLMARK_WNT_BETA_CATENIN_SIGNALING	HALLMARK_WNT_BETA_CATENIN_SIGNALING	42	-0.485	-1.473	0.042	0.022	0.479
HALLMARK_P53_PATHWAY	HALLMARK_P53_PATHWAY	197	-0.378	-1.406	0.023	0.043	0.716
HALLMARK_HEME_METABOLISM	HALLMARK_HEME_METABOLISM	195	-0.357	-1.323	0.048	0.088	0.937
HALLMARK_FATTY_ACID_METABOLISM	HALLMARK_FATTY_ACID_METABOLISM	155	-0.341	-1.242	0.108	0.158	0.989
HALLMARK_REACTIVE_OXIGEN_SPECIES_PATHWAY	HALLMARK_REACTIVE_OXIGEN_SPECIES_PATHWA	47	-0.388	-1.193	0.229	0.212	0.999
HALLMARK_BILE_ACID_METABOLISM	HALLMARK_BILE_ACID_METABOLISM	112	-0.344	-1.191	0.185	0.208	0.999
HALLMARK_CHOLESTEROL_HOMEOSTASIS	HALLMARK_CHOLESTEROL_HOMEOSTASIS	73	-0.340	-1.107	0.322	0.330	1.000
HALLMARK_UV_RESPONSE_UP	HALLMARK_UV_RESPONSE_UP	154	-0.282	-1.030	0.426	0.467	1.000
HALLMARK_PANCREAS_BETA_CELLS	HALLMARK_PANCREAS_BETA_CELLS	40	-0.325	-0.984	0.485	0.544	1.000
HALLMARK_PEROXISOME	HALLMARK_PEROXISOME	101	-0.234	-0.808	0.796	0.872	1.000
HALLMARK_KRAS_SIGNALING_DN	HALLMARK_KRAS_SIGNALING_DN	192	-0.187	-0.702	0.973	0.980	1.000
HALLMARK_PROTEIN_SECRETION	HALLMARK_PROTEIN_SECRETION	96	-0.162	-0.561	0.996	0.997	1.000

Supplementary Table 2:

		Number of patients	*% patients*	*Number with metastases*	*% metastases*	*MFS p-value*
Total		441	100.0%	173	39.2%	
Age	≤50	93	21.1%	36	38.7%	0.90 (NS)
	>50	348	78.9%	137	39.4%	
SBR histological grade	I	57	13.2%	11	19.3%	0.0017
	II	219	50.7%	88	40.2%	
	III	156	36.1%	70	44.9%	
Lymph node status	0	116	26.5%	35	30.2%	<0.0001
	1-3	229	52.4%	79	34.5%	
	>3	92	21.1%	57	62.0%	
Macroscopic tumor size	≤25mm	216	49.9%	65	30.1%	<0.0001
	>25mm	217	50.1%	107	49.3%	
ERα status	Negative	113	25.6%	46	40.7%	0.19 (NS)
	Positive	328	74.4%	127	38.7%	
PR status	Negative	188	42.6%	81	43.1%	0.036
	Positive	253	57.4%	92	36.4%	
ERBB2 status	Negative	350	79.4%	135	38.6%	0.49 (NS)
	Positive	91	20.6%	38	41.8%	
Molecular subtypes	HR- HER2-	67	15.2%	25	37.3%	0.17 (NS)
	HR- HER2+	41	9.3%	20	48.8%	
	HR+ HER2-	283	64.2%	110	38.9%	
	HR+ HER2+	50	11.3%	18	36.0%	

Supplementary Table 2: Pathological and clinical characteristics of patients in relation to metastasis free survival (MFS). ERα: oestrogen receptor alpha; PR: progesterone receptor; ERBB2: human epidermal growth factor receptor 2; HR: hormone receptor. MFS: metastasis-free survival. P values are calculated with the Log-rank test. NS: not significant.

Supplementary Table 3

Characteristics		HR	95% CI	*p*-value
PLK1	≤5.35	1		
	>5.35	2.29	1.51-3.48	0.00011
Macroscopic tumour size	≤25mm	1		0.0034
	>25mm	1.61	1.17-2.22	
Lymph node status	0	1		<0.0001
	1-3	1.61	1.29-2.01	
	>3	2.59	1.66-4.04	
SBR histological grade	I	1		0.85
	II	1.03	0.79-1.33	
	III	1.05	0.63-1.76	
PR status	Positive	1		0.42
	Negative	1.14	0.83-1.58	

Supplementary Table 3: Multivariate COX analysis of MFS for PLK1 expression level in the series of 441 breast cancers. HR: hazard ratio. 95%CI: 95% Confidential Interval. P values were calculated by the multivariate COX analysis. PLK1 cut-off levels 5.35, hazard ratio, 2.29; 95% confidence interval 1.51-3.48; tumour size cut-off 25 mm, hazard ratio, 1.61; 95% confidence interval, 11.17-2.22; lymph node status 1-3, hazard ratio, 1.61; 95% confidence interval 11.29-2.01; lymph node status >3, hazard ratio, 2.59; 95% confidence interval 1.66-4.04.
