# Supplementary material for: PLK1 inhibition exhibits strong anti-tumoral activity in CCND1-driven breast cancer metastases with acquired palbociclib resistance
Source: Nat Commun. 2020 Aug 13;11:4053. doi: 10.1038/s41467-020-17697-1 (PMC7426966; doi:10.1038/s41467-020-17697-1)
Supplement: Supplementary file 2 — Description of Additional Supplementary Files [file 41467_2020_17697_MOESM2_ESM.docx]

**Description of Additional Supplementary Files**

File Name: Supplementary Data 1

Description: Targeted NGS analysis of PDX

File Name: Supplementary Data 2

Description: copy number alterations in patients' and PDX tumours

File Name: Supplementary Data 3

Description: Statistical analysis of in vivo experiments

File Name: Supplementary Data 4

Description: Statistical analysis of RT-PCR experiments

File Name: Supplementary Data 5

Description: RIME raw data
